# Product Authentication Using Two Mitochondrial Markers Reveals Inconsistent Labeling and Substitution of Canned Tuna Products in the Taiwanese Market

**DOI:** 10.3390/foods10112655

**Published:** 2021-11-02

**Authors:** Chia-Hao Chang, Yueh-Tzu Kao, Ting-Ting Huang, Yu-Chun Wang

**Affiliations:** 1 TIGP Biodiversity Program, Tunghai University, No.1727, Sec.4, Taiwan Boulevard, Xitun District, Taichung City 407224, Taiwan; chiahao0928@gmail.com (C.-H.C.); miaotingting00@gmail.com (T.-T.H.); 2 Department of Life Science, Tunghai University, No.1727, Sec.4, Taiwan Boulevard, Xitun District, Taichung City 407224, Taiwan; hyakkiin@gmail.com; 3 Planning and Information Division, Fisheries Research Institute, No.199, Hou-Ih Road, Keelung City 202008, Taiwan

**Keywords:** Thunnini, substitution, mislabeling rate, one species-one name, adulteration

## Abstract

Fish of the tribe Thunnini represent a significant proportion of the stock caught by the fishing industry, with many of these fishes being collectively called tuna. However, only certain species can be used legally as an ingredient in canned tuna products, depending on regional food regulations. In Taiwan, only *Thunnus* species or *Katsuwonus pelamis* can be used as canned tuna. Here, we authenticated 90 canned tuna products, including 25 cat food samples, by sequencing two mitochondrial regions, 16S rRNA (16S) and the control region (CR). BLAST analysis revealed that *Sarda orientalis*, *Euthynnus affinis*, *Auxis rochei*, and *Auxis thazard* are all used as substitutes for legitimate tuna products. We found that 63.33% of investigated samples are true canned tuna, i.e., contain *Thunnus* species or skipjack tuna. We advocate that the Taiwanese government publishes an official standardized list of fishes, especially so that scientific, Chinese and vernacular names can be assigned unambiguously based on a “one species-one name policy”, thereby clarifying which species can be used in seafood products such as tuna. Furthermore, we feel that the large-scale and long-term monitoring of canned tuna products is warranted to fully assess the extent of tuna product adulteration in Taiwan.

## 1. Introduction

Approximately 17% of the global human population’s intake of animal protein in 2017 constituted fish [1]. Although aquaculture satisfied about half of that consumption, wild-capture from oceans, lakes and rivers remains a mainstay of the global fishing industry. Among these wild-caught fishes, scombrids are particularly important fishery resources, especially species in the tribe Thunnini that constitute ~10% of the international seafood market [2,3]. In 2018, the global catch of Thunnini species represented ~7.9 million tons, 58% of which can be attributed to skipjack tuna (*Katsuwonus pelamis*) (in Chinese: 正鰹) and yellowfin tuna (*Thunnus albacares*) (in Chinese: 黃鰭鮪) [1]. A large proportion of the Thunnini catch is destined for the canning industry [4,5].

The tribe Thunnini comprises five genera: *Thunnus* (in Chinese: 鮪屬), *Katsuwonus* (in Chinese: 正鰹屬), *Auxis* (in Chinese: 花鰹屬), *Euthynnus* (in Chinese: 巴鰹屬), and *Allothunnus* (in Chinese: 細鰹屬). Fishes of this tribe can be generally termed “tuna”. For example, *Auxis rochei* is called bullet tuna (in Chinese: 圓花鰹), *Euthynnus alleteratus* is little tunny (in Chinese: 小巴鰹), and *Allothunnus fallai* is slender tuna (in Chinese: 細鰹). However, the Chinese translation of tuna is 鮪 in Taiwan or 金槍魚 in Mainland China, which specifically refers solely to *Thunnus* spp. Previously, many different scombrids were used as an ingredient in “canned tuna”, even if they did not belong to the tribe Thunnini. For instance, *Sarda* (in Chinese: 齒鰆屬) spp. were once widely used in canned tuna because they possess a similar taste and texture to it [6]. Importantly, a species of the tribe Thunnini may not always be used legally as a canned tuna product ingredient. Various legislative bodies have developed regulations that clearly define which species can be used in canned tuna products (Table 1). The Food and Agriculture Organization (FAO) and the federal government of the United States allow spotted tuna to be used as canned tuna, but that species is prohibited by Taiwanese and Japanese regulations. In general, fishes of the genus *Thunnus* and skipjack tuna are widely recognized as legal canned tuna species. To align with international standards, the Taiwan Food and Drug Administration allows skipjack tuna to be used in 鮪 (for tuna)-labeled canned tuna products, even though it does not belong to the genus *Thunnus*, but other “pseudo-tunas” can no longer be used legally as a canned tuna ingredient.

Seafood mislabeling is profuse worldwide [12,13,14,15,16,17,18,19,20,21]. Such mislabeling can be categorized into two types, i.e., deliberate or unintentional. Deliberate mislabeling primarily involves the substitution of high-priced fishes with low-priced ones for financial reasons, though the reverse scenario also arises occasionally, perhaps due to illegal fishing. Unintentional mislabeling occurs when morphologically similar fishes are misidentified, when the usage of vernacular names is not unified, or when product information is lost along the supply chain. Whatever the form of mislabeling, it ultimately entails consumer deception, public health risk, problems for fisheries management, and has religious implications (reviewed in Chang et al. [22]).

Traditional morphology-based identification is rarely applied to seafood because many products undergo physical (e.g., filleting) or chemical (e.g., smoking) processing before being sold. These aspects of food processing typically eliminate diagnostic morphological characters needed for species authentication. Fortunately, molecular authentication based on nucleic acid sequence similarity can overcome this limitation. DNA can be obtained from a tiny piece of tissue and it is more resistant to degradation and food processing. Therefore, DNA-based authentication is being widely employed to identify species in seafood [15,21,23,24,25,26,27,28,29,30]. 

The increasing global popularity of Japanese cuisine has markedly increased market demand for tuna, since *Thunnus* fishes are important elements of sashimi and sushi. The development of freezing technology and booming global trade in the early 1970s has transformed the bluefin tunas (*T. thynnus* (in Chinese: 大西洋黑鮪), *T. maccoyii* (in Chinese: 南方黑鮪), and *T. orientalis* (in Chinese: 太平洋黑鮪)) from a cat food into a delicacy served at high-end restaurants [31]. Bluefin tunas are the most sought after of all *Thunnus* fishes, attaining the largest size and greatest price. However, increased consumption has also threatened their stocks, which are decreasing and the status of all three species is deemed Critical (IUCN). Today, regional fishery management organizations are responsible for managing and monitoring tuna fishing in order to keep it sustainable [32].

The soaring demand for *Thunnus* fishes, especially bluefin tuna, makes them very vulnerable to mislabeling. Previous molecular authentication studies on sushi reported that many fishes are used as substitutes for *Thunnus* species, including escolar (*Lepidocybium flavobrunneum*) (in Chinese: 鱗網帶鰆), salmon (*Salmo salar*) (in Chinese: 安大略鱒), banded rudderfish (*Seriola zonata*) (in Chinese: 環帶鰤), great amberjack (*Seriola dumerili*) (in Chinese: 杜氏鰤), skipjack tuna, little tunny, as well as various shark species [14,17,18,20,33,34,35,36,37]. Furthermore, the value of different *Thunnus* species varies, prompting high-priced bluefin tuna or bigeye tuna (*T. obesus*) (in Chinese: 大目鮪) to be substituted for a cheaper species such as yellowfin tuna. Notably, enforcement of fishery management can drive reverse substitution, whereby high-priced bluefin tuna is sold as cheap yellowfin tuna or *Thunnus* fishes are labeled as skipjack tuna to enable market entry of illegal catch [33,38,39].

Although DNA-based methods are very powerful tools for authenticating fish products, food processing, and especially canning, can limit their applicability. To date, conventional DNA barcoding remains the most widely deployed authentication approach, whereby a ~650-bp region of the mitochondrial gene encoding for cytochrome c subunit I (COI) is sequenced as a bioidentification “barcode” [40,41]. However, the high heat treatment integral to the canning process largely degrades DNA into small fragments [42,43], so shorter fragments (or nested polymerase chain reaction, PCR) must be deployed for canned products [4,5,6,39,44,45], but a comprehensive investigation of canned tuna substitution in a particular region has not yet been conducted. In Taiwan, the mislabeling rate of tuna products varies according to the product type. Chang et al. [22] documented that all tuna-labeled meals produced at conveyor-belt sushi restaurants appear to truly come from *Thunnus* fishes, but Xiong et al. [46] and Hwang et al. [44] detected mislabeled Taiwan canned tuna products. Therefore, the goal of this study is to estimate levels of canned tuna product adulteration and to determine which species of scombrids are being marketed as canned product in the Taiwanese market.

## 2. Materials and Methods

### 2.1. Sample Collection

We purchased a total of 90 canned tuna products, belonging to 59 brands, from grocery stores or online, encompassing all major brands in Taiwan. Twenty-five of the collected samples represented canned cat food. Cans were selected if their label showed the Chinese word 鮪 (for tuna), if the company website claimed the product was made from *Thunnus* fishes, if the ingredients list contained *Thunnus* spp. or skipjack tuna, or if an image on the label indicated the can harbored *Thunnus* fishes. We recorded information, typically written in Chinese, on brand, manufacturer or importer, place of manufacture, labeling, and ingredients. If the cans were imported from the USA or Japan, the respective English or Japanese labels were also recorded (Table 2). The sampled cans were first photographed using a smartphone (Supplementary Information S1), and then a small quantity of the contents of each can was removed using autoclaved dissection tools, washed with 95% ethanol, before being preserved in 99.5% ethanol at −20 °C until DNA extraction. Some of the canned cat food products contained more than one type of meat, so potential *Thunnus* meat was selected based on color and texture.

### 2.2. Molecular Identification

DNA was extracted from each of the 90 tissue samples using DNA Extraction Kit S (Cat No./ID: GS100, Geneaid). PCR amplifications of the mitochondrial 16S rRNA fragment (16S) (85 bp) were performed in a mixture containing 5 ng template DNA, 12.5 μL of 2× Taq PCR MasterMix (GN-PCR201-01, Genomix), and 12.5 μmol of each forward and reverse primer. We used primers designed by Horreo et al. [47] and modified them by adding M13 primer to facilitate sequencing: Forward, M13F(-20)16S-HF (5′-GTA AAA CGA CGG CCA GTA TAA CAC GAG AAG ACC CT-3′); Reverse, M13R(-24)16S-HR1+2 (5′-AACAGCTATGACCATGCCCRCGGTCGCCCCA AC-3′). These primers were made up to a final volume of 25 μL using distilled water. If BLAST analysis (in the NCBI basic local alignment search tool) indicated that a sequenced 16S fragment belonged to *Thunnus* spp., we PCR-amplified a fragment of the mitochondrial control region (CR, approximately 236 bp) from the same DNA sample. CR amplification was conducted in a mixture containing 5 ng template DNA, 12.5 μL of 2× Taq PCR MasterMix (GN-PCR201-01, Genomix), and 12.5 μmol of each forward and reverse primer—Forward, Tuna-CR_F; Reverse, Tuna-CR_R1 or Tuna-CR_R2 [48]—made up to a final volume of 25 μL using distilled water. Thermal cycling began with one cycle at 95 °C for 4 min, followed by 35 cycles of denaturation at 95 °C for 30 s, 47–55 °C for 30 s, and 72 °C for 30 s and, finally, a single extension step at 72 °C for 7 min. PCR products were purified using a PCR DNA Fragment Extraction Kit (Geneaid, Taipei, Taiwan). The amplified mitochondrial fragments were subjected to Sanger sequencing, performed by Mission Biotech. (Taipei, Taiwan) using M13 sequencing primers. Primer sequences linked to the amplified fragments were trimmed before constructing the contigs using CodonCode Aligner. The mitochondrial sequences we generated in this study have not been submitted to GenBank as they do not come from voucher specimens.

### 2.3. Data Analysis

Edited 16S sequences were first aligned using ClustalW in MEGA11 [49], and then the haplotypes were determined in DnaSP 6. Species identity for each 16S haplotype was achieved by comparing them (by BLAST) to reference sequences in the NCBI GenBank database. Following the approach of both Armani et al. [50] and Horreo et al. [51], only matches displaying full sequence coverage and 100% similarity, and with unambiguous species-level scientific names, were considered positive fish identifications. If more than one fish species was shown as a positive match, all of them were considered potential candidates (Table 2). 

All CR sequences used in the study of Mitchell and Hellberg (2016) were downloaded to serve as reference sequences, and then our CR fragments and reference sequences were aligned in ClustalW. A neighbor-joining (NJ) analysis was then conducted based on Kimura two-parameter (K2P) distances and 1 × 10^3^ bootstrapping replicates in MEGA11 [49]. According to the phylogenetic species concept [52], monophyly is a prerequisite for species recognition, so our specimens were authenticated based on the reference species with which they clustered and formed a monophyletic group (with high statistical support, i.e., bootstrapping value ≥ 70) in the NJ phenogram.

### 2.4. Comparison of Analytical Results and Product Labels

We compared the molecular identification of each sample to the ingredient list of the sampled cans. Since a Taiwanese government-approved standard for common names of fishes does not exist, the English names of labeled Chinese names were ascertained from the fish database of Taiwan (https://fishdb.sinica.edu.tw/ (accessed on 26 October 2021)). Although the Chinese symbols 鰹魚could broadly refer to any species from the genera *Auxis* (in Chinese: 花鰹屬), *Euthynnus* (in Chinese: 巴鰹屬), and *Katsuwonus* (in Chinese: 正鰹屬), skipjack tuna is the only species generally termed 鰹魚 and that can be used legally in Taiwan to make canned tuna (Table 1). Therefore, we assumed that if 鰹魚 appeared on the ingredient list of a canned tuna product, it specifically represented skipjack tuna. Many of the imported products displayed labeling in Chinese and the language of source, but in those cases we exclusively relied on the Chinese label since Chinese is the only official language in Taiwan. 

A sample was judged as displaying inconsistent labeling if the fish name in the ingredient list could not be linked unambiguously to a *Thunnus* species or skipjack tuna. It was then deemed mislabeled if the molecularly authenticated species it contained did not match the ingredient list on the label. Where a vernacular name used in an ingredient list refers to more than one species, a case of mislabeling was assigned when the molecularly authenticated species did not correspond to any fishes bearing that vernacular name. Finally, we determined a product as being true canned tuna if it contained *Thunnus* species or skipjack tuna.

## 3. Results

**Figure 1:**
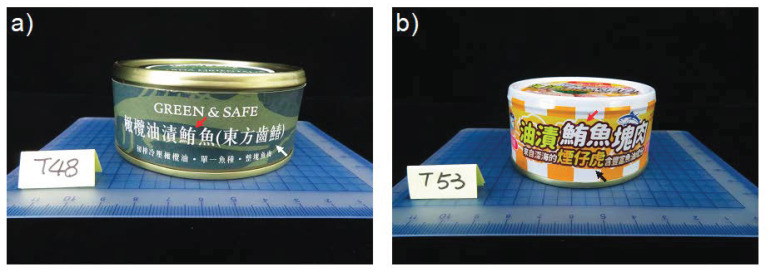
Taiwanese canned tuna products displaying inconsistent labeling. “鮪” (red arrows) in the Chinese labels declares both of these canned tuna products as legally being made from *Thunnus* fishes or skipjack tuna (*Katsuwonus pelamis*) (in Chinese: 正鰹). (**a**) Oriental bonito (*Sarda orientalis*) (in Chinese: 東方齒鰆) (white arrow) in the label indicates the product contains that species. (**b**) The Chinese vernacular name 煙仔虎 (black arrow) may represent both oriental bonito and skipjack tuna.

We observed inconsistent labeling in 11 of 65 canned tuna products destined for human consumption, but no such problem with cat food products. Inconsistent labeling reflected canned tuna products also claiming to be made from oriental bonito (*Sarda orientalis*) (in Chinese: 東方齒鰆) or displaying the ambiguous vernacular name 煙仔虎, which can refer to either skipjack tuna or oriental bonito (Figure 1, Table 2).

We successfully amplified the 16S fragment from all 90 samples, resulting in eight haplotypes (Supplementary Information S2). All haplotypes could be identified to species-level by BLAST analysis, but only haplotypes Hap_A and Hap_E specifically relate to oriental bonito and skipjack tuna, respectively. More than one species was identified by BLAST analysis for the remaining six haplotypes, but based on the number of BLAST hits we assume Hap_B represents kawakawa (*Euthynnus affinis*) (in Chinese: 巴鰹), Hap_C is skipjack tuna, Hap_D and Hap_F are *Thunnus* species, Hap_H is either longtail tuna (*T. tonggol*) (in Chinese: 長腰鮪) or bigeye tuna (*T. obesus*), and Hap_G is either bullet tuna or frigate tuna (*Auxis thazard*) (in Chinese: 扁花鰹).

**Figure 2:**
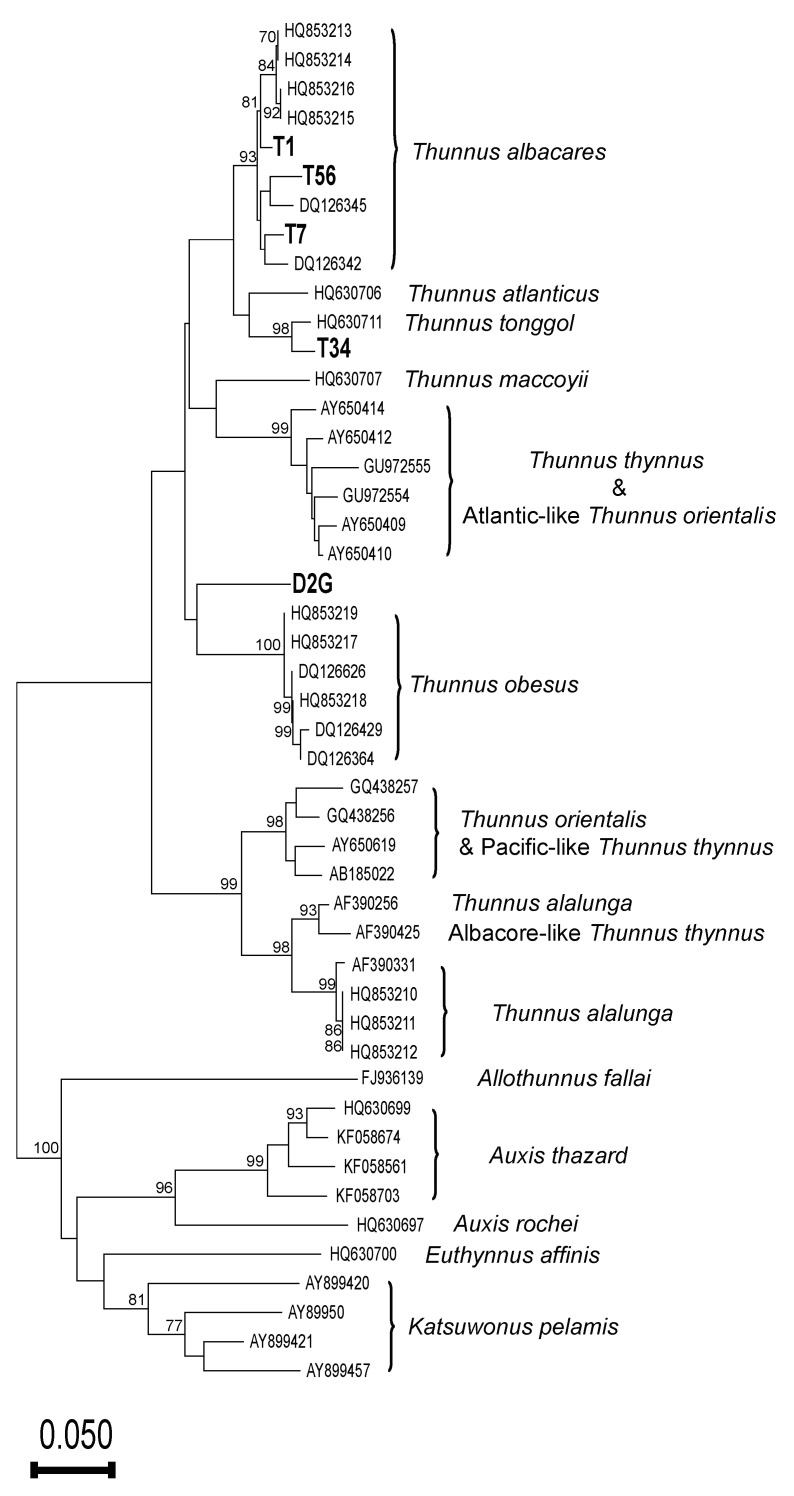
Neighbor-joining (NJ) tree of the K2P model of 47 taxa inferred from 256 bp of mitochondrial control region (CR) sequences with 1000 bootstrapping replicates. Each terminal is labeled with the GenBank accession number or sample code. Bootstrapping values ≥ 70 are indicated on the respective branches.

Our BLAST analysis of 16S sequences revealed that 31 of our samples contained *Thunnus* fishes. However, the success rate of CR amplification from those samples was quite low (5/31; 16%). The aligned CR dataset (Supplementary Information S3) for NJ analysis is 256 bp in length and contains 47 taxa, including 159 variable sites and 131 parsimony-informative sites. The NJ analysis of CR sequences supports that samples T1, T7, and T56 are yellowfin tuna, and that sample T34 is longtail tuna, but we could not authenticate sample D2G based on its CR sequence (Figure 2).

Excluding the canned cat food samples that were all accurately labeled, 25 of the remaining 65 canned tuna products were mislabeled and a further three were potential mislabeling cases. Our BLAST analysis confirmed that sample T36 contained *Thunnus* fish, but did not reveal which species. We found the labeling of sample T48 to be misleading. In Chinese, the symbol “鮪” (for tuna) is never associated with oriental bonito, so it is unreasonable for the symbol for oriental bonito to be placed in parentheses following “鮪魚” (representing “tuna fish”) on the label for this sample. We observed a similar issue for sample T4, since neither skipjack tuna nor oriental bonito can be regarded as a type of “鮪” (*Thunnus* spp.). Since the ingredient statement on the label fails to clearly indicate which species is contained in the can, it is difficult to judge whether or not these two samples are mislabeled. Notably, many of the products identified as exhibiting inconsistent labeling were also found to be mislabeled. The mislabeling rate of canned products for human consumption was 38% (25/65). Mislabeling was even more pervasive among cat food products, with a rate of 68% (17/25). The main reason for this high mislabeling rate of cat food products is that many claim to contain *Thunnus* fishes but are in actual fact made from skipjack tuna. The overall mislabeling rate for the 90 tested products of this study is ~47% (42/90).

Of the 65 human food products we tested, 37 (57%) legitimately contained either *Thunnus* fishes or skipjack tuna, and 20 out of 25 cat food products are true canned tuna. Overall, the proportion of true canned tuna products is about 63.33% (57/90) in this study.

## 4. Discussion

According to Article 28 of the Act Governing Food Safety and Sanitation in Taiwan, public labeling, promotion and advertisement of foods or food additives, cleansers, utensils, containers or packaging designated by the central competent authority shall not be false, exaggerated or misleading. The 11 cases of inconsistent labeling we identified among our 90 samples, which display “鮪” (for tuna) on their labels but also list scombrids other than *Thunnus* species or skipjack tuna as an ingredient, obviously mislead customers into believing these products contain true canned tuna. For this study, we solely relied on the information on Chinese labeling, but we also noted conflicting information between Chinese and source-language labeling of imported products. For example, the ingredient statement in Japanese of sample T42 clearly declares that it is made from albacore tuna (*T. alalunga*), but its Chinese label only states that it contains *Thunnus* fishes (in Chinese: 鮪魚). Similarly, the Japanese label of sample T59 indicates skipjack tuna as an ingredient (in Japanese: かつお), but its Chinese label specifies yellowfin tuna (in Chinese: 黃鰭鮪) (Table 2). Such conflicting labeling of imported products not only confuses consumers but may also circumvent legal controls.

The “one species-one name” policy is critical to the authentication of fishery products [38]. Clearly, usage of scientific names could enable investigators to easily judge if a product is mislabeled. Under European Union labeling regulations, including the species’ scientific name on fishery product labels is mandatory [53]. However, scientific names are not required on Taiwanese fishery products nor are such names familiar to the public. Xiong et al. [54] and Chang et al. [22] advocated the adoption of the Chinese-Latin Dictionary of Fish Names (https://fishdb.sinica.edu.tw/chi/chinesequer1.php (accessed on 26 October 2021)) as a standard list of fishes in Chinese corresponding to scientific nomenclature. This Dictionary indeed clarifies that the Chinese symbols 東方齒鰆 (in English: oriental bonito) correspond to *Sarda orientalis*, but it does not include other Chinese vernacular names. Thus, any official “one species-one name” standard should not only contain scientific and Chinese names, but also incorporate vernacular names.

Notably, our success rates for amplifying the two mitochondrial DNA fragments differed considerably—100% for 16S, but only 16% for CR. The canning process is known to damage DNA molecules, with Quinteiro et al. [43] documenting that most DNA segments extracted from canned tuna are <100 bp in length. Therefore, it is not surprising that amplification of the 85-bp 16S region was more successful than the 236-bp CR fragment (Binominal Generalized Logical Model, *p* < 0.01).

Apart from haplotypes Hap_A and Hap_C, a single species was not identified by BLAST for the other 16S haplotypes. There are a number of possible reasons for that outcome. First, DNA degradation through the canning process limits molecular authentication based on longer sequences, such as via conventional DNA barcoding. Accordingly, shorter DNA segments must be targeted, but they contain less information and so are less likely to unambiguously assign a specific species [55]. Second, molecular identification based on mitochondrial sequences is very sensitive to gene flow and incomplete lineage sorting [25,56,57]. The tribe *Thunnus* comprises very closely related species, some of which display genetic introgression [58,59]. Consequently, though Hap_D, Hap_E, and Hap_H are all clearly form *Thunnus* fishes, their exact species identity remains unclear. Though conventional DNA barcoding can distinguish *Thunnus* fishes [60,61,62], it would be problematic to amplify the ~650 bp barcode from the degraded DNA of canned samples. Third, a reliable database is crucial to accurate DNA-based identification [63]. GenBank does not guarantee that deposited sequences display correct species names. For example, the BLAST result for Hap_C matches multiple sequences for skipjack tuna sequences and one for yellowfin tuna (GenBank accession number: KM055376), implying that accession KM055376 is very likely misidentified. Hence, as highlighted in a number of studies [22,64,65], a reliable and complete DNA reference database for authenticating seafood resources is sorely needed. 

In this study, we found that many canned tuna products in Taiwan are made from oriental bonito, kawakawa, bullet tuna, or frigate tuna instead of legitimate *Thunnus* fishes or skipjack tuna. Although oriental bonito was never found in canned cat food products, the other three substituted fishes were identified in both human and cat food samples. These same four species have also been reported as illegitimate tuna substitutes in other studies [44,46,48,50,66,67]. Though istiophorid fishes have been reported as mislabeled *Thunnus* products in other studies [46,68], we did not detect them in this study.

Our NJ analysis of CR sequences, including five haplotypes generated in this study, further revealed that both yellowfin tuna and longtail tuna are used in canned tuna products. Yellowfin tuna is one of the commonest canned tunas [5,69], so it is not surprising that three of our five CR haplotypes clustered with yellowfin tuna sequences (sample T34 was identified as longtail tuna, and sample D2G could not be identified to species level). Our difficulties with amplifying the CR region mean that the specific *Thunnus* composition of canned tuna products in Taiwan remains unclear. Identifying canned tuna products to species level is important because certain *Thunnus* fishes have higher mercury levels [70], posing a human health risk. Therefore, mitochondrial regions other than CR, such as ATP synthase membrane subunit 8 (ATP8), ATP6, and COIII could be considered [71], or smaller CR fragments could be targeted. 

We observed the terms 白身鮪魚 or 鮪魚白肉 commonly in the ingredient statements of our cat food samples (Table 2), reflecting the high mislabeling rate (17/25) among cat food products. However, most of the cat food samples (20/25) still represented true canned tuna, albeit not the species that might be expected. To date, there is no official definition for either of these two Chinese terms. They may be translated as “light tuna”, which often refers to yellowfin tuna or skipjack tuna, but could actually be any fishes mentioned in the Code of Federal Regulation Title 21 (CFR 161.190) and with flesh color in the Munsell color system ≥5.3 [48]. If those terms were to be officially recognized as translations of light tuna, then the mislabeling rate of cat food we report herein would be much lower (down to 8/25) (Binominal Generalized Logical Model, *p* < 0.01). Accordingly, we implore the responsible authorities to clearly define the terms for use in canned product labeling.

Although we found that 63.33% of our samples are true canned tuna, this outcome may not reflect the actual adulteration level of canned tuna products in Taiwan. First, we selected only one small piece of tissue from each can, but the mixing of tuna species in cans has been found in the European market [4]. An assessment of how prevalent the mixing of tuna species is in cans in the Taiwanese market would be needed to determine how close our calculated adulteration level is to the real scenario. Second, we solely sampled major brands, so there are some that remain to be assessed, especially of cat food. Moreover, seasonality in scombrid catch may alter the species composition of adulterated canned tuna products. Thus, more comprehensive and long-term monitoring of the species composition of canned tuna products is needed.

## 5. Conclusions

We report an overall mislabeling rate of 46.67% among the 90 samples of this study, with 63.33% of sampled canned products being true canned tuna legitimately made from *Thunnus* fish or skipjack tuna. In many cases, the labels of the sampled canned tuna products would confuse customers as to what species they contain. Either they contain species such as oriental bonito that do not conform to Taiwanese legislation, or ill-defined terms such as 白身鮪魚 or Chinese vernacular names are used. We assert that a standard list of scientific names and their corresponding Chinese and vernacular names conforming to the “one species-one name” principle, as well as clear definitions of terms for use in canned tuna labeling, is crucial to tackling fish product adulteration. We found that ~37% of investigated canned tuna products contain illegitimate species. In many cases, the manufacturers have substituted so-called “pseudo-tunas”, such as oriental bonito, kawakawa, bullet tuna, and frigate tuna, for legal species, i.e., *Thunnus* species and skipjack tuna. Our study demonstrates that a pair of primers targeting a short segment (85 bp) of 16S performs well in amplifying DNA extracted from canned food samples. However, the limited information content provided by this short sequence hampered molecular identification to species level, especially given the close phylogenetic relationships and potential for gene flow among *Thunnus* species. Moreover, the CR fragment we targeted largely proved uninformative, likely owing to the extreme DNA fragmentation caused by high heat treatment during the canning process. Our previous study of seafood adulteration in conveyor-belt sushi restaurants revealed no case of tuna fraud in such establishments [22], so such adulteration appears to be more common in canned products. A large-scale and long-term monitoring study would help fully establish the extent of canned tuna fraud in the Taiwanese market.

## Figures and Tables

**Table 1:** Scientific, English common, Chinese common and vernacular names of scombrid fishes permitted by various legislative bodies as canned tuna or bonito products.

Scientific Name	English Common Name	Chinese Name	Chinese Vernacular Name	Taiwan ^1^	FAO ^2^	USA ^3^	Japan ^4^	European Union ^5^
Thunnini tribe								
*Thunnus alalunga*	Albacore tuna	長鰭鮪	串仔、長鰭串、白肉串、長鬚甕串	✓	✓	✓	✓	✓
*Thunnus albacares*	Yellowfin tuna	黃鰭鮪	串仔、黃奇串、黑肉、甕串、黃鰭金槍魚、黃鰭串	✓	✓	✓	✓	✓
*Thunnus atlanticus*	Blackfin tuna	黑鰭鮪		✓	✓	✓		✓
*Thunnus obesus*	Bigeye tuna	大目鮪	大目仔、大眼鮪、大目串、短墩、串仔、短鮪	✓	✓	✓	✓	✓
*Thunnus maccoyii*	Southern bluefin tuna	南方黑鮪		✓	✓	✓		✓
*Thunnus thynnus*	Atlantic bluefin tuna	大西洋黑鮪		✓	✓	✓		✓
*Thunnus orientalis*	Pacific bluefin tuna	太平洋黑鮪	黑鮪、黑甕串、黑暗串、東方鮪、東方藍鰭鮪、黑串、金槍魚、烏甕串、串魚、魚因、烏暗串	✓	✓	✓	✓	✓
*Thunnus tonggol*	Longtail tuna	長腰鮪	黑鰭串、串仔、長實、長翼、小黃鰭鮪	✓	✓	✓	✓	✓
*Katsuwonus pelamis*	Skipjack tuna	正鰹	煙仔虎、煙仔、小串、柴魚、肥煙、鯤、煙仔魚、卓鮶、大煙	✓	✓	✓	✓	✓
*Auxis rochei*	Bullet tuna	圓花鰹	煙管仔、竹棍魚、槍管煙、鎗管煙子			✓		
*Auxis thazard*	Frigate tuna	扁花鰹	煙仔魚、油煙、花煙、平花鰹、憨煙、平花煙、腩肚煙			✓		
*Euthynnus affinis*	Kawakawa	巴鰹	三點仔、煙仔、倒串、鯤、花煙、大憨煙、花鰋		✓	✓		
*Euthynnus alleteratus*	Little tunny	小巴鰹			✓	✓		
*Euthynnus lineatus*	Black skipjack tuna	黑巴鰹			✓	✓		
*Allothunnus fallai*	Slender tuna	細鰹				✓		
Sardini tribe								
*Sarda orientalis*	Oriental bonito	東方齒鰆	煙仔虎、梳齒、西齒、疏齒、掠齒煙、烏鰡串、西齒煙		✓			
*Sarda sarda*	Atlantic bonito	齒鰆			✓			
*Sarda chilensis*	Eastern Pacific bonito	智利齒鰆			✓			

^1^ 107年度『建構完整食品標示管理體系計畫』—『宣稱鮪魚罐頭之標示說明會』, Taiwan; ^2^ Standard for canned tuna and bonito CXS 70-1981, Codex Alimentarius FAO-WHO; ^3^ Code of federal regulations CFR 21. Sec. 161.190. United State Food and Drug Administration, USA; ^4^ 水産物缶詰及び水産物瓶詰の日本農林規格, Japan; ^5^ European regulation (Council Regulation (EEC) No 1536/92) [7,8,9,10,11].

**Table 2:** List of all canned tuna product samples authenticated by 16S rRNA BLAST and the results of neighbor-joining (NJ) analysis based on the mitochondrial control region (CR).

No.	Sample Code	Brand	Manufacturer or Importer	Place of Manufacture	Chinese Label	English Label	Declared Ingredient	English Translation of Ingredient	Inconsistent Labeling	*16S rRNA* Haplotype Code	16S BLAST (No. of Hits)	CR NJ Analysis	Mislabeled	True Canned Tuna
1	T1	遠洋牌	興毅冷凍食品	Taiwan	鮪魚片	Light tuna in oil	鮪魚	*Thunnus* spp.	NO	Hap_F	*Thunnus tonggol* (101), *T. orientalis* (4), *T. atlanticus* (2), *T. thynnus* (4), *T. albacares* (14), *T. maccoyii* (6), *T. obesus* (4), *T. alalunga* (1), *Katsuwonus pelamis* (1)	*T. albacares*	NO	YES
2	T53	遠洋牌	興毅冷凍食品	Taiwan	油漬鮪魚肉塊(煙仔虎)		煙仔虎	Skipjack tuna or oriental bonito	YES	Hap_G	*Auxis thazard* (6)*, A. rochei* (7)*, Euthynnus affinis* (1)		YES	NO
3	T57	遠洋牌	興毅冷凍食品	Taiwan	玉米+鮪魚	Tuna + sweet corn	鮪魚	*Thunnus* spp.	NO	Hap_F	*Thunnus tonggol* (101), *T. orientalis* (4), *T. atlanticus* (2), *T. thynnus* (4), *T. albacares* (14), *T. maccoyii* (6), *T. obesus* (4), *T. alalunga* (1), *Katsuwonus pelamis* (1)		NO	YES
4	T62	三興	惠眾食品	Taiwan	紅SH油漬鮪魚(東方齒鰆)	Tuna in oil	東方齒鰆	Oriental bonito	YES	Hap_A	*Sarda orientalis* (3)		NO	NO
5	T2	三興	惠眾食品	Taiwan	SH水煮鮪魚(東方齒鰆)	Tuna in brine	東方齒鰆	Oriental bonito	YES	Hap_A	*Sarda orientalis* (3)		NO	NO
6	T3	好媽媽	東和食品	Taiwan	纖麗鮪魚	Tuna flakes in brine	鮪魚	*Thunnus* spp.	NO	Hap_C	*Katsuwonus pelamis* (7), *Thunnus albacares* (1)		YES	YES
7	T52	好媽媽	東和食品	Taiwan	辣妹鮪魚	Tuna flakes in chili oil	鮪魚	*Thunnus* spp.	NO	Hap_F	*Thunnus tonggol* (101), *T. orientalis* (4), *T. atlanticus* (2), *T. thynnus* (4), *T. albacares* (14), *T. maccoyii* (6), *T. obesus* (4), *T. alalunga* (1), *Katsuwonus pelamis* (1)		NO	YES
8	T4	好媽媽	東和食品	Taiwan	三明治鮪魚 (煙仔虎)	Tuna sandwich	煙仔虎 (鮪魚)	Skipjact tuna or oriental bonito (*Thunnus* spp.)	YES	Hap_A	*Sarda orientalis* (3)		?	NO
9	T49	好媽媽	東和食品	Taiwan	無添加玉米鮪魚 Corn Tuna	Corn tuna	煙仔虎	Skipjact tuna or oriental bonito	YES	Hap_A	*Sarda orientalis* (3)		NO	NO
10	T18	蘇澳區漁會	東和食品	Taiwan	水煮鮪魚	Canned boiled tuna	鮪魚	*Thunnus* spp.	NO	Hap_B	*Euthynnus affinis* (4), *E. lineatus* (1)		YES	NO
11	T20	冬山河	東和食品	Taiwan	三明治鮪魚		鮪魚	*Thunnus* spp.	NO	Hap_B	*Euthynnus affinis* (4), *E. lineatus* (1)		YES	NO
12	T21	鮮拚鮮	東和食品	Vietnam	鮪魚罐頭		鮪魚	*Thunnus* spp.	NO	Hap_G	*Auxis thazard* (6)*, A. rochei* (7)*, Euthynnus affinis* (1)		YES	NO
13	T5	紅鷹牌	活寶食品	Taiwan	紅鷹牌海底雞(魚罐)		鮪魚	*Thunnus* spp.	NO	Hap_C	*Katsuwonus pelamis* (7), *Thunnus albacares* (1)		YES	YES
14	T6	紅鷹牌	活寶食品	Taiwan	紅鷹牌海底雞水煮(魚罐)		東方齒鰆	Oriental bonito	YES	Hap_A	*Sarda orientalis* (3)		NO	NO
15	T7	紅鷹牌	活寶食品	Taiwan	海底雞鮮の味片狀(魚罐)		鮪魚 (鮪屬)	*Thunnus* spp. (*Thunnus*)	NO	Hap_F	*Thunnus tonggol* (101), *T. orientalis* (4), *T. atlanticus* (2), *T. thynnus* (4), *T. albacares* (14), *T. maccoyii* (6), *T. obesus* (4), *T. alalunga* (1), *Katsuwonus pelamis* (1)	*T. albacares*	NO	YES
16	T8	紅鷹牌	活寶食品	Taiwan	幼筍鮪魚	Bamboo shoots tuna	正鰹 (鮪族)	Skipjack tuna (Thunnini)	NO	Hap_G	*Auxis thazard* (6)*, A. rochei* (7)*, Euthynnus affinis* (1)		YES	NO
17	T9	紅鷹牌	活寶食品	Taiwan	海底雞鮮の味塊狀(魚罐)		鮪魚	*Thunnus* spp.	NO	Hap_A	*Sarda orientalis* (3)		YES	NO
18	T54	紅鷹牌	活寶食品	Taiwan	紅鷹牌海底雞鮪魚片	Slices tuna	正鰹 (鮪族)	Skipjack tuna (Thunnini)	NO	Hap_A	*Sarda orientalis* (3)		YES	NO
19	T55	紅鷹牌	活寶食品	Taiwan	洋蔥鮪魚	Onion tuna	正鰹 (鮪族)	Skipjack tuna (Thunnini)	NO	Hap_G	*Auxis thazard* (6)*, A. rochei* (7)*, Euthynnus affinis* (1)		YES	NO
20	T10	台糖	台糖	Taiwan	香筍鮪魚	Tuna flakes with bamboo shoots	鮪魚、鰹魚	*Thunnus* spp., skipjack tuna	NO	Hap_F	*Thunnus tonggol* (101), *T. orientalis* (4), *T. atlanticus* (2), *T. thynnus* (4), *T. albacares* (14), *T. maccoyii* (6), *T. obesus* (4), *T. alalunga* (1), *Katsuwonus pelamis* (1)		NO	YES
21	T46	台糖	台糖	Taiwan	台糖三明治鮪魚(油漬)	Tuna flakes in oil	鮪鰹魚類	*Thunnus* spp., skipjack tuna	NO	Hap_H	*Thunnus tonggol* (1), *T. obesus* (1)		NO	YES
22	T61	台糖	台糖	Taiwan	台糖鮪魚片(油漬)	Tuna flakes in oil	鮪魚、鰹魚	*Thunnus* spp., skipjack tuna	NO	Hap_F	*Thunnus tonggol* (101), *T. orientalis* (4), *T. atlanticus* (2), *T. thynnus* (4), *T. albacares* (14), *T. maccoyii* (6), *T. obesus* (4), *T. alalunga* (1), *Katsuwonus pelamis* (1)		NO	YES
23	T11	新東陽	新東陽	Taiwan	新東陽水煮鮪魚片		鮪魚、鰹魚	*Thunnus* spp., skipjack tuna	NO	Hap_G	*Auxis thazard* (6)*, A. rochei* (7)*, Euthynnus affinis* (1)		YES	NO
24	T12	愛之味	愛之味	Taiwan	愛之味鮪魚片	Tuna slice	鮪魚	*Thunnus* spp.	NO	Hap_F	*Thunnus tonggol* (101), *T. orientalis* (4), *T. atlanticus* (2), *T. thynnus* (4), *T. albacares* (14), *T. maccoyii* (6), *T. obesus* (4), *T. alalunga* (1), *Katsuwonus pelamis* (1)		NO	YES
25	T45	愛之味	愛之味	Thailand	珍寶三明治鮪魚	AGV deli style tuna	鮪魚	*Thunnus* spp.	NO	Hap_F	*Thunnus tonggol* (101), *T. orientalis* (4), *T. atlanticus* (2), *T. thynnus* (4), *T. albacares* (14), *T. maccoyii* (6), *T. obesus* (4), *T. alalunga* (1), *Katsuwonus pelamis* (1)		NO	YES
26	T13	老船長	金春勝食品	Taiwan	老船長特製鮪魚(煙仔虎)	Tuna fish	煙仔虎	Skipjact tuna or oriental bonito	YES	Hap_A	*Sarda orientalis* (3)		NO	NO
27	T56	老船長	金春勝食品	Taiwan	筍仔鮪魚	Tuna flakes with bamboo shoots	鮪魚	*Thunnus* spp.	NO	Hap_F	*Thunnus tonggol* (101), *T. orientalis* (4), *T. atlanticus* (2), *T. thynnus* (4), *T. albacares* (14), *T. maccoyii* (6), *T. obesus* (4), *T. alalunga* (1), *Katsuwonus pelamis* (1)	*T. albacares*	NO	YES
28	T14	新宜興	隆育企業	Taiwan	水煮鮪魚	Tuna in brine	鮪魚、鰹魚	*Thunnus* spp., skipjack tuna	NO	Hap_H	*Thunnus tonggol*, *T. obesus*		NO	YES
29	T47	新宜興	隆育企業	Taiwan	新宜興三明治鮪魚	Tuna sandwich	鮪鰹魚類	*Thunnus* spp., skipjack tuna	NO	Hap_G	*Auxis thazard* (6)*, A. rochei* (7)*, Euthynnus affinis* (1)		YES	NO
30	T58	新宜興	隆育企業	Taiwan	新宜興原味鮪魚片	Tuna slice	鮪魚、鰹魚	*Thunnus* spp., skipjack tuna	NO	Hap_G	*Auxis thazard* (6)*, A. rochei* (7)*, Euthynnus affinis* (1)		YES	NO
31	T15	Viridis Vivus	隆育企業	Taiwan	V V 鮪魚片	Tuna slice	鮪魚、鰹魚	*Thunnus* spp., skipjack tuna	NO	Hap_G	*Auxis thazard* (6)*, A. rochei* (7)*, Euthynnus affinis* (1)		YES	NO
32	T16	同榮	同榮實業	Taiwan	同榮鮪魚片	Tuna flake in oil	煙仔虎	Skipjact tuna or oriental bonito	YES	Hap_A	*Sarda orientalis* (3)		NO	NO
33	T44	同榮	同榮實業	Vietnam	三明治特餐		鰹魚	Skipjack tuna	NO	Hap_B	*Euthynnus affinis* (4), *E. lineatus* (1)		YES	NO
34	T17	爭鮮	爭鮮	Taiwan	油漬鮪魚	Tuna flakes in oil	鮪魚、鰹魚	*Thunnus* spp., skipjack tuna	NO	Hap_H	*Thunnus tonggol* (1), *T. obesus* (1)		NO	YES
35	T19	藍海洋	旺來興	Taiwan	三明治鮪魚	Tuna in oil	鮪魚、鰹魚	*Thunnus* spp., skipjack tuna	NO	Hap_G	*Auxis thazard* (6)*, A. rochei* (7)*, Euthynnus affinis* (1)		YES	NO
36	T22	KY	寬元行(進口商)	Vietnam	三明治鮪魚	Shredded light tuna in oil	鮪魚、鰹魚	*Thunnus* spp., skipjack tuna	NO	Hap_F	*Thunnus tonggol* (101), *T. orientalis* (4), *T. atlanticus* (2), *T. thynnus* (4), *T. albacares* (14), *T. maccoyii* (6), *T. obesus* (4), *T. alalunga* (1), *Katsuwonus pelamis* (1)		NO	YES
37	T23	大海鮪魚	力遠貿易	Vietnam	鮪魚罐頭		鮪魚	*Thunnus* spp.	NO	Hap_F	*Thunnus tonggol* (101), *T. orientalis* (4), *T. atlanticus* (2), *T. thynnus* (4), *T. albacares* (14), *T. maccoyii* (6), *T. obesus* (4), *T. alalunga* (1), *Katsuwonus pelamis* (1)		NO	YES
38	T24	紅龍	碁富食品	Thailand	紅龍嚴選三明治鮪魚		鮪魚	*Thunnus* spp.	NO	Hap_C	*Katsuwonus pelamis* (7), *Thunnus albacares* (1)		YES	YES
39	T25	金熊	洋鼎(進口商)	Indonesia	金熊三明治鮪魚		鮪魚	*Thunnus* spp.	NO	Hap_C	*Katsuwonus pelamis* (7), *Thunnus albacares* (1)		YES	YES
40	T27	MACORO	寬元行(進口商)	Vietnam	每口樂片狀三明治鮪魚	Tuna flake in oil	鮪魚、鰹魚	*Thunnus* spp., skipjack tuna	NO	Hap_H	*Thunnus tonggol* (1), *T. obesus* (1)		NO	YES
41	T28	南海洋	力遠貿易	Taiwan	油漬鮪魚(煙仔虎)		煙仔虎	Skipjact tuna or oriental bonito	YES	Hap_A	*Sarda orientalis* (3)		NO	NO
42	T29	California Fresh	Unicord Public	Thailand	California Fresh油漬鮪魚片	Skipjack tuna shredded in soybean oil	鮪鰹魚類 (正鰹)	*Thunnus* spp., skipjack tuna (skipjack tuna)	NO	Hap_C	*Katsuwonus pelamis* (7), *Thunnus albacares* (1)		NO	YES
43	T30	慶全	老三林食品	Taiwan	慶全油漬鮪魚		鮪鰹魚肉	*Thunnus* spp., skipjack tuna	NO	Hap_A	*Sarda orientalis* (3)		YES	NO
44	T40	老三林	老三林食品	Taiwan	油漬魚(煙仔虎)		煙仔虎	Skipjact tuna or oriental bonito	YES	Hap_A	*Sarda orientalis* (3)		NO	NO
45	T31	三乃	三乃	Taiwan	三乃油漬鮪魚片肉	light meat tuna	鮪魚	*Thunnus* spp.	NO	Hap_G	*Auxis thazard* (6)*, A. rochei* (7)*, Euthynnus affinis* (1)		YES	NO
46	T32	雄雞標	駿伸企業*	Thailand	雄雞標特級初榨橄欖油浸鮪魚片 Omega3	Tuna omega3 in extra virgin olive oil	精選鮪魚	*Thunnus* spp.	NO	Hap_F	*Thunnus tonggol* (101), *T. orientalis* (4), *T. atlanticus* (2), *T. thynnus* (4), *T. albacares* (14), *T. maccoyii* (6), *T. obesus* (4), *T. alalunga* (1), *Katsuwonus pelamis* (1)		NO	YES
47	T33	雄雞標	駿伸企業*	Thailand	雄雞標初榨橄欖油鮪魚塊	Tuna chunks in extra virgin olive oil	精選鮪魚	*Thunnus* spp.	NO	Hap_F	*Thunnus tonggol* (101), *T. orientalis* (4), *T. atlanticus* (2), *T. thynnus* (4), *T. albacares* (14), *T. maccoyii* (6), *T. obesus* (4), *T. alalunga* (1), *Katsuwonus pelamis* (1)		NO	YES
48	T36	大鯖魚夢工廠	山萬海產加工廠	Taiwan	黑鮪魚罐頭	Bluefin tuna	黑鮪魚肉	bluefin tuna	NO	Hap_F	*Thunnus tonggol* (101), *T. orientalis* (4), *T. atlanticus* (2), *T. thynnus* (4), *T. albacares* (14), *T. maccoyii* (6), *T. obesus* (4), *T. alalunga* (1), *Katsuwonus pelamis* (1)		?	YES
49	T37	美鷹牌	康律企業(代理商)	Thailand	美鷹牌鮪魚		鰹魚、鮪魚	*Thunnus* spp., skipjack tuna	NO	Hap_F	*Thunnus tonggol* (101), *T. orientalis* (4), *T. atlanticus* (2), *T. thynnus* (4), *T. albacares* (14), *T. maccoyii* (6), *T. obesus* (4), *T. alalunga* (1), *Katsuwonus pelamis* (1)		NO	YES
50	T41	丸漢堡	寶丸食品	Vietnam	寶丸鮪魚罐頭		鮪魚	*Thunnus* spp.	NO	Hap_B	*Euthynnus affinis* (4), *E. lineatus* (1)		YES	NO
51	T43	慶祥	慶祥食品	Taiwan	慶祥黑鮪魚罐頭		鮪鰹魚類	*Thunnus* spp., skipjack tuna	NO	Hap_F	*Thunnus tonggol* (101), *T. orientalis* (4), *T. atlanticus* (2), *T. thynnus* (4), *T. albacares* (14), *T. maccoyii* (6), *T. obesus* (4), *T. alalunga* (1), *Katsuwonus pelamis* (1)		NO	YES
52	T48	GERRN&SAFE	永豐生技	Taiwan	橄欖油漬鮪魚(東方齒鰆)	Sarda orientalis in extra virgin olive oil	鮪魚 (東方齒鰆)	*Thunnus* spp. (oriental bonito)	YES	Hap_A	*Sarda orientalis* (3)		?	NO
53	T42	Kirkland signature	Costco	Fiji	kirkland signature科克蘭鮪魚罐頭	Albacore	Chinese: 鮪魚/English: Albacore tuna	Chinese: *Thunnus* spp./English: albacore tuna	NO	Hap_D	*Thunnus obesus* (3), *T. thynnus* (2), *T. albacares* (2), *T. alalunga* (3), *T. orientalis* (1)		NO	YES
54	T26	マルハ	マルハニチロ株式会社	Japan	丸哈鮪魚罐	Tuna in soy sauce	Chinese: 金槍魚/Japanese: まぐろ	Chinese and Japanese: *Thunnus* spp.	NO	Hap_F	*Thunnus tonggol* (101), *T. orientalis* (4), *T. atlanticus* (2), *T. thynnus* (4), *T. albacares* (14), *T. maccoyii* (6), *T. obesus* (4), *T. alalunga* (1), *Katsuwonus pelamis* (1)		NO	YES
55	T50	良好生活	くらし良好	Thailand	生活良好鮪魚罐(三入)		Chinese: 鮪魚/Japanese: きはだまぐろ	Chinese: *Thunnus* spp./Japanese: yellowfin tuna	NO	Hap_F	*Thunnus tonggol* (101), *T. orientalis* (4), *T. atlanticus* (2), *T. thynnus* (4), *T. albacares* (14), *T. maccoyii* (6), *T. obesus* (4), *T. alalunga* (1), *Katsuwonus pelamis* (1)		NO	YES
56	T51	黃金口福(コウフク)	KODANML GROUP CO., LTD	Thailand	黃金口福油三入漬鮪魚罐	Light tuna	Chinese: 鮪魚/Japanese: まぐろ	Chinese and Japanese: *Thunnus* spp.	NO	Hap_B	*Euthynnus affinis* (4), *E. lineatus* (1)		YES	NO
57	T59	HOTEI	ホテイフーズ	Thailand	HOTEI油漬鮪魚罐頭		Chinese: 黃鰭鮪/Japanese: かつお	Chinese: yellowfin tuna/Japanese: skipjack tuna	NO	Hap_C	*Katsuwonus pelamis* (7), *Thunnus albacares* (1)		YES	YES
58	T65	HOTEi	ホテイフーズ	Japan	豪德水煮鮪魚罐		Chinese: 黃鮪魚/Japanese: きはだまぐろ	Chinese and Japanese: yellowfin tuna	NO	Hap_F	*Thunnus tonggol* (101), *T. orientalis* (4), *T. atlanticus* (2), *T. thynnus* (4), *T. albacares* (14), *T. maccoyii* (6), *T. obesus* (4), *T. alalunga* (1), *Katsuwonus pelamis* (1)		NO	YES
59	T60	今津	今津株式会社	Thailand	今津鰹魚玉米罐		Chinese: 鰹魚/Japanese: きはだまぐろ	Chinese: skipjack tuna/Japanese: yellowfin tuna	NO	Hap_F	*Thunnus tonggol* (101), *T. orientalis* (4), *T. atlanticus* (2), *T. thynnus* (4), *T. albacares* (14), *T. maccoyii* (6), *T. obesus* (4), *T. alalunga* (1), *Katsuwonus pelamis* (1)		YES	YES
60	T34	極洋	極洋株式會社	Thailand	極洋鮪魚罐頭-油漬		Chinese: 鮪魚/Japanese: まぐろ	Chinese and Japanese: *Thunnus* spp.	NO	Hap_F	*Thunnus tonggol* (101), *T. orientalis* (4), *T. atlanticus* (2), *T. thynnus* (4), *T. albacares* (14), *T. maccoyii* (6), *T. obesus* (4), *T. alalunga* (1), *Katsuwonus pelamis* (1)	*T. albacares*	NO	YES
61	T35	極洋	極洋株式會社	Thailand	極洋油漬鰹魚罐		Chinese: 鮪魚/Japanese: まぐろ	Chinese and Japanese: *Thunnus* spp.	NO	Hap_C	*Katsuwonus pelamis* (7), *Thunnus albacares* (1)		YES	YES
62	T38	稻葉	いなば食品	Thailand	稻葉鮪魚鰹魚罐	Light tuna	Chinese: 鮪魚、鰹魚/Japanese: まぐろ	Chinese: *Thunnus* spp. and skipjack tuna/Japanese: *Thunnus* spp.	NO	Hap_C	*Katsuwonus pelamis* (7), *Thunnus albacares* (1)		NO	YES
63	T39	伊藤	伊藤食品	Japan	伊藤油漬鮪魚(金罐)		Chinese: 鮪魚/Japanese: まぐろ	Chinese and Japanese: *Thunnus* spp.	NO	Hap_F	*Thunnus tonggol* (101), *T. orientalis* (4), *T. atlanticus* (2), *T. thynnus* (4), *T. albacares* (14), *T. maccoyii* (6), *T. obesus* (4), *T. alalunga* (1), *Katsuwonus pelamis* (1)		NO	YES
64	T63	Hagoromo	はごろもフーズ株式会社	Japan	一本釣頂級鮪魚罐		Chinese: 鮪魚/Japanese: びんながまぐろ	Chinese: *Thunnus* spp./Japanese: albacore tuna	NO	Hap_F	*Thunnus tonggol* (101), *T. orientalis* (4), *T. atlanticus* (2), *T. thynnus* (4), *T. albacares* (14), *T. maccoyii* (6), *T. obesus* (4), *T. alalunga* (1), *Katsuwonus pelamis* (1)		NO	YES
65	T64	SSK	清水食品株式会社	Japan	油漬鮪魚		Chinese: 鮪魚/Japanese: きはだまぐろ	Chinese: *Thunnus* spp./Japanese: yellowfin tuna	NO	Hap_F	*Thunnus tonggol* (101), *T. orientalis* (4), *T. atlanticus* (2), *T. thynnus* (4), *T. albacares* (14), *T. maccoyii* (6), *T. obesus* (4), *T. alalunga* (1), *Katsuwonus pelamis* (1)		NO	YES
**Canned cat food**														
66	B1B	SEEDS	THAI UNION	Thailand	Hello Fresh 好鮮原汁湯罐 (清蒸鮪魚)	Tuna	鮪魚	*Thunnus* spp.	NO	Hap_C	*Katsuwonus pelamis* (7), *Thunnus albacares* (1)		YES	YES
67	B1C	SEEDS	UNICORD	Thailand	Tuna愛貓天然食(兩倍鮮嫩雞肉+白身鮪魚)	Chicken & Tuna light meat	白身鮪魚、雞肉	*Thunnus* spp., chicken	NO	Hap_C	*Katsuwonus pelamis* (7), *Thunnus albacares* (1)		YES	YES
68	B2E	SEEDS	UNICORD	Thailand	Tuna愛貓天然食	Chicken & tuna light meat	雞肉、白身鮪魚	Chicken, *Thunnus* spp.	NO	Hap_C	*Katsuwonus pelamis* (7), *Thunnus albacares* (1)		YES	YES
69	B2F	SEEDS	UNICORD	Thailand	Tuna愛貓天然食	Light tuna meat & shirasu	白身鮪魚、吻仔魚	Whitebait, *Thunnus* spp.	NO	Hap_C	*Katsuwonus pelamis* (7), *Thunnus albacares* (1)		YES	YES
70	B2G	SEEDS	THAI UNION	Thailand	Bistro Cat 特級銀貓健康餐罐	Tuna light meat + shrimp	白身鮪魚、蝦肉	*Thunnus* spp., shrimp	NO	Hap_C	*Katsuwonus pelamis* (7), *Thunnus albacares* (1)		YES	YES
71	B1D	YAMI亞米	Hi-Q Food	Thailand	健寶 鮪魚蟹柳活力餐		鮪魚、蟹柳	*Thunnus* spp., crab stick	NO	Hap_B	*Euthynnus affinis* (4), *E. lineatus* (1)		YES	NO
72	B3A	GOEN	Pataya Food	Thailand	御宴湯罐 白身鮪魚+雞肉		白身鮪魚、雞肉	*Thunnus* spp., chicken	NO	Hap_C	*Katsuwonus pelamis* (7), *Thunnus albacares* (1)		YES	YES
73	B3B	GOEN	Pataya Food	Thailand	御宴湯罐 白身鮪魚+鮭魚		白身鮪魚、鮭魚	*Thunnus* spp., salmon	NO	Hap_C	*Katsuwonus pelamis* (7), *Thunnus albacares* (1)		YES	YES
74	B3F	元氣家族	Pataya Food	Thailand	元氣家族金罐 鮪魚+鯛魚		鮪魚、鯛魚	*Thunnus* spp., snapper	NO	Hap_B	*Euthynnus affinis* (4), *E. lineatus* (1)		YES	NO
75	B3H	元氣家族	Pataya Food	Thailand	元氣家族金罐 鮪魚+鮮蝦		鮪魚、鮮蝦	*Thunnus* spp., shrimp	NO	Hap_B	*Euthynnus affinis* (4), *E. lineatus* (1)		YES	NO
76	C1A	愛情貴族	UNICORD	Thailand	CIH-C08白身鮪魚&牛肉		白身鮪魚、牛肉	*Thunnus* spp., beef	NO	Hap_E	*Katsuwonus pelamis* (1)		YES	YES
77	C1B	愛情貴族	UNICORD	Thailand	CIH-C02白身鮪魚&吻仔魚		白身鮪魚、吻仔魚	*Thunnus* spp., whitebait	NO	Hap_C	*Katsuwonus pelamis* (7), *Thunnus albacares* (1)		YES	YES
78	C2B	每日貓罐	泛美力	Taiwan	每日貓罐-鮪魚+蟹味絲 湯罐		鮪魚、蟹味絲	*Thunnus* spp., crab stick	NO	Hap_F	*Thunnus tonggol* (101), *T. orientalis* (4), *T. atlanticus* (2), *T. thynnus* (4), *T. albacares* (14), *T. maccoyii* (6), *T. obesus* (4), *T. alalunga* (1), *Katsuwonus pelamis* (1)		NO	YES
79	C2C	每日貓罐	泛美力	Taiwan	每日貓罐-鮪魚+巴沙魚 湯罐		鮪魚、巴沙魚	*Thunnus* spp., basa fish	NO	Hap_D	*Thunnus obesus* (3), *T. thynnus* (2), *T. albacares* (2), *T. alalunga* (3), *T. orientalis* (1)		NO	YES
80	C4A	鼎食貓罐	沅慶企業有限公司	Taiwan	鼎食貓罐{新鮮鮪魚+丁香魚}		鮪魚、丁香魚	*Thunnus* spp., clove fish	NO	Hap_F	*Thunnus tonggol* (101), *T. orientalis* (4), *T. atlanticus* (2), *T. thynnus* (4), *T. albacares* (14), *T. maccoyii* (6), *T. obesus* (4), *T. alalunga* (1), *Katsuwonus pelamis* (1)		NO	YES
81	C4C	鼎食貓罐	沅慶企業有限公司	Taiwan	鼎食貓罐{新鮮鮪魚+櫻花蝦}		鮪魚、櫻花蝦	*Thunnus* spp., sakura shrimp	NO	Hap_F	*Thunnus tonggol* (101), *T. orientalis* (4), *T. atlanticus* (2), *T. thynnus* (4), *T. albacares* (14), *T. maccoyii* (6), *T. obesus* (4), *T. alalunga* (1), *Katsuwonus pelamis* (1)		NO	YES
82	C5A	怪獸部落	Songkla Canning	Thailand	無膠無穀鮮肉煲-鮪魚片湯罐	Flaked tuna in broth	鮪魚	*Thunnus* spp.	NO	Hap_C	*Katsuwonus pelamis* (7), *Thunnus albacares* (1)		YES	YES
83	D1B	YAMI亞米	Hi-Q Food Products Co	Thailand	鮮鮪.雞肉白金大餐	YAMI Platinum	鮪魚白肉	*Thunnus* spp.	NO	Hap_G	*Auxis thazard* (6)*, A. rochei* (7)*, Euthynnus affinis* (1)		YES	NO
84	D1C	YAMI亞米	Hi-Q Food Products Co	Thailand	鮮鮪.青花魚.蟹柳白金大餐	YAMI Platinum	鮪魚白肉	*Thunnus* spp.	NO	Hap_G	*Auxis thazard* (6)*, A. rochei* (7)*, Euthynnus affinis* (1)		YES	NO
85	D2B	TRIL GY	Real Pet Food Company	Thailand	奇境 無穀貓罐 野生鮪魚燉雞湯		鮪魚	*Thunnus* spp.	NO	Hap_C	*Katsuwonus pelamis* (7), *Thunnus albacares* (1)		YES	YES
86	D2C	O’KAT	黑逗國際有限公司	Thailand	美喵人生 無榖化毛餐		鮪魚	*Thunnus* spp.	NO	Hap_C	*Katsuwonus pelamis* (7), *Thunnus albacares* (1)		YES	YES
87	D2G	Rico	喬泰寵物用品企業有限公司	Taiwan	芮可-貓用副食鮮湯罐2號(鮪雞+鰹魚)		鮪魚、雞肉、鰹魚	*Thunnus* spp., chicken, skipjack tuna	NO	Hap_F	*Thunnus tonggol* (101), *T. orientalis* (4), *T. atlanticus* (2), *T. thynnus* (4), *T. albacares* (14), *T. maccoyii* (6), *T. obesus* (4), *T. alalunga* (1), *Katsuwonus pelamis* (1)	*T. tonggol*	NO	YES
88	E1E	樂妙貓	サスナ株式会社	Japan	樂妙貓3號-鮪.吻仔		Chinese: 鮪魚、吻仔魚/Japanese: マグロ、しらす	Chinese and Japanese: *Thunnus* spp., whitebait	NO	Hap_F	*Thunnus tonggol* (101), *T. orientalis* (4), *T. atlanticus* (2), *T. thynnus* (4), *T. albacares* (14), *T. maccoyii* (6), *T. obesus* (4), *T. alalunga* (1), *Katsuwonus pelamis* (1)		NO	YES
89	E2C	厚肉肉	悠遊國際實業股份有限公司	Taiwan	T.N.A悠遊厚肉肉主食罐-一品鮪魚拚鮭魚		鮪魚、鮭魚	*Thunnus* spp., salmon	NO	Hap_F	*Thunnus tonggol* (101), *T. orientalis* (4), *T. atlanticus* (2), *T. thynnus* (4), *T. albacares* (14), *T. maccoyii* (6), *T. obesus* (4), *T. alalunga* (1), *Katsuwonus pelamis* (1)		NO	YES
90	E2D	愛喜雅	サスナ株式会社	Japan	燒津43號-鮪.雞.鮭		Chinese: 鮪魚、雞肉、鮭魚/Japanese: マグロ、鶏ササミ、紅鮭	Chinese and Japanese: *Thunnus* spp., chicken, salmon	NO	Hap_F	*Thunnus tonggol* (101), *T. orientalis* (4), *T. atlanticus* (2), *T. thynnus* (4), *T. albacares* (14), *T. maccoyii* (6), *T. obesus* (4), *T. alalunga* (1), *Katsuwonus pelamis* (1)		NO	YES

## Data Availability

The data presented in this study are available on request from the first author.

## Supplementary Materials

The following are available online at https://www.mdpi.com/article/10.3390/foods10112655/s1, Supplementary Information S1: The photos of all sampled canned tuna items, Supplementary Information S2: The 16S haplotypes, Supplementary Information S3: The aligned CR sequences for constructing NJ phylogenetic analysis.
