# Supplementary material for: Product Authentication Using Two Mitochondrial Markers Reveals Inconsistent Labeling and Substitution of Canned Tuna Products in the Taiwanese Market
Source: Foods. 2021 Nov 2;10(11):2655. doi: 10.3390/foods10112655 (PMC8623642; doi:10.3390/foods10112655)
Supplement: Supplementary file 1 [file foods-10-02655-s001.zip › foods-1391712-supplementary.pdf]

Supplementary S1

T1	T2	T3	T4
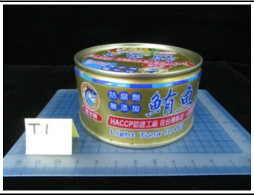	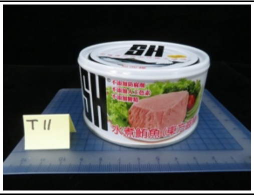	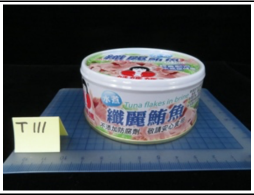	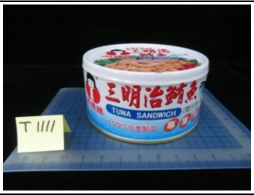
T5	T6	T7	T8
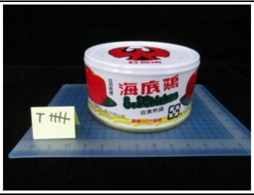	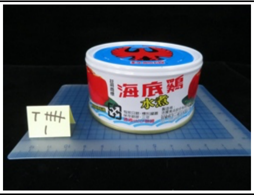	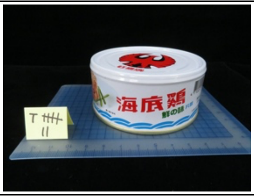	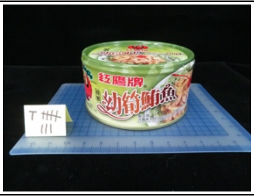
T9	T10	T11	T12
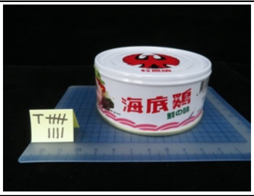	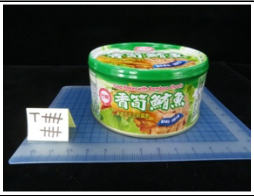	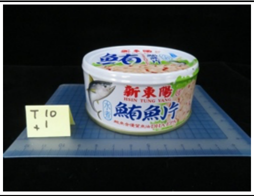	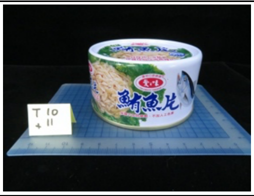
T13	T14	T15	T16
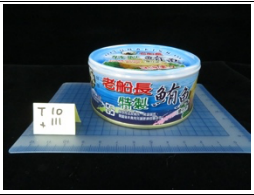	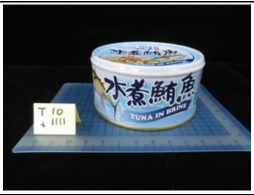	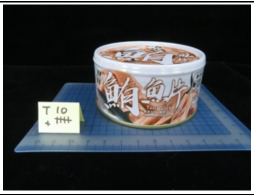	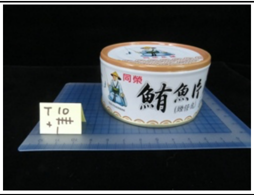
T17	T18	T19	T20
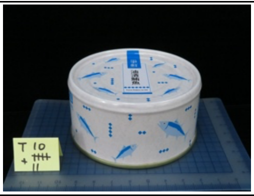	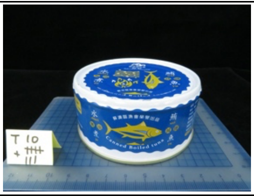	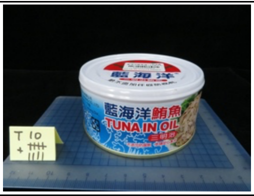	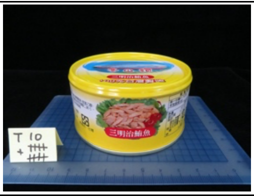
T21	T22	T23	T24
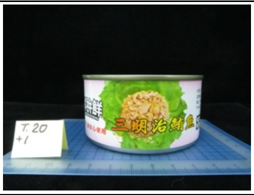	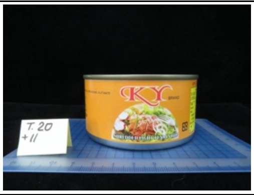	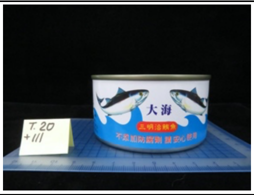	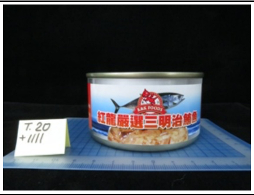
T25	T26	T27	T28
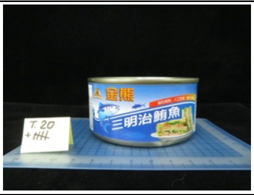	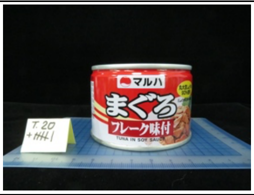	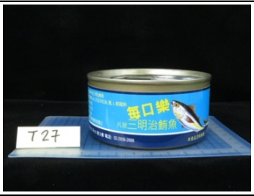	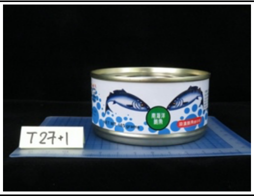

T29	T30	T31	T32
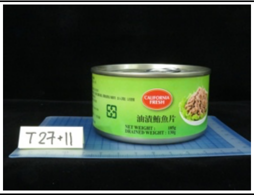	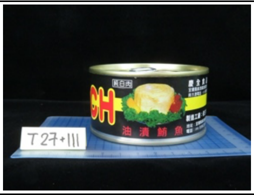	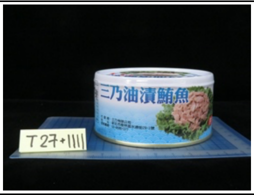	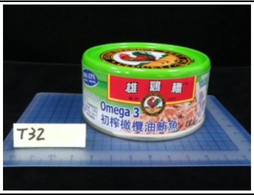
T33	T34	T35	T36
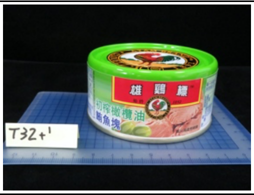	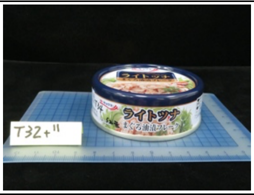	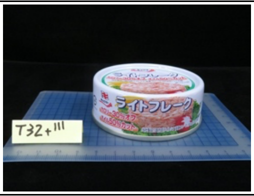	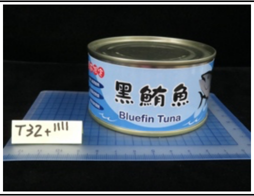
T37	T38	T39	T40
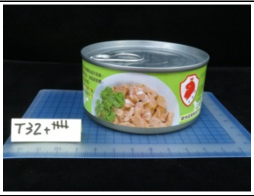	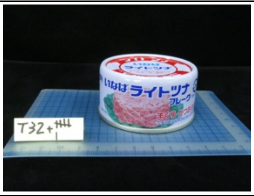	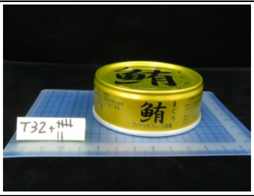	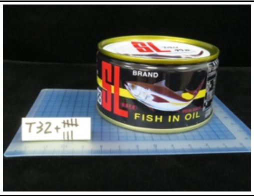
T41	T42	T43	T44
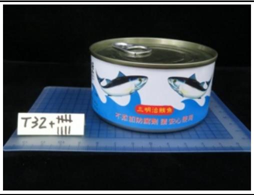	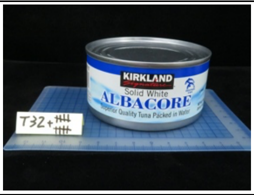	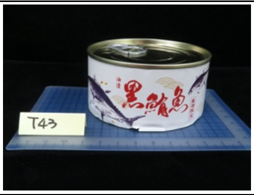	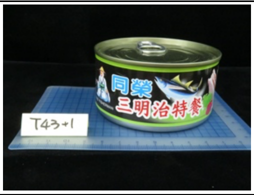
T45	T46	T47	T48
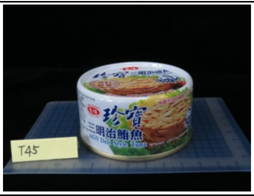	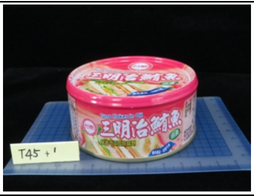	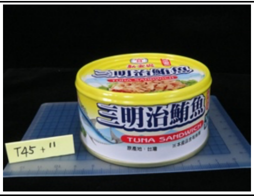	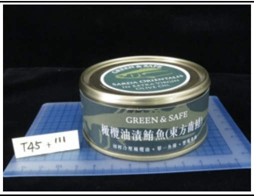
T49	T50	T51	T52
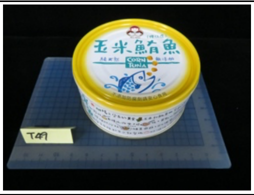	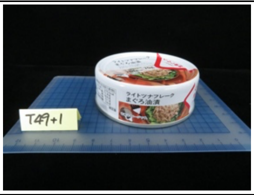	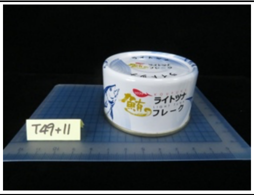	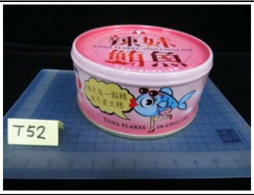
T53	T54	T55	T56
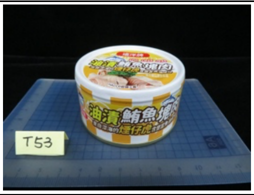	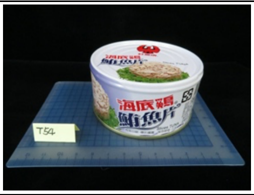	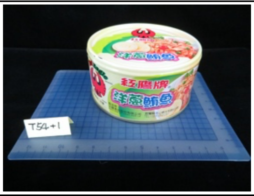	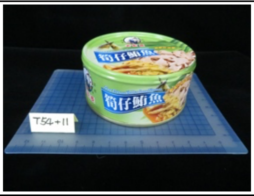
T57	T58	T59	T60

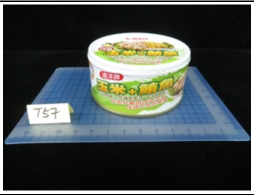	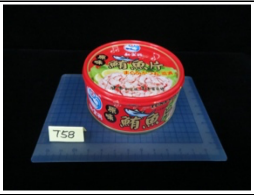	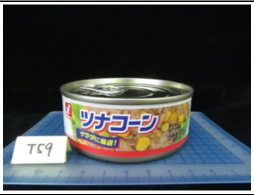	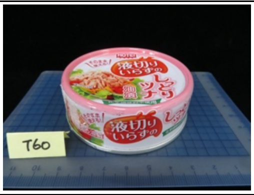
T61	T62	T63	T64
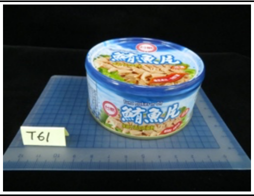	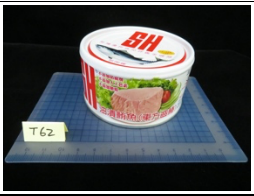	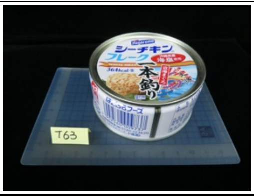	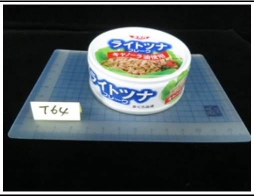
T65			
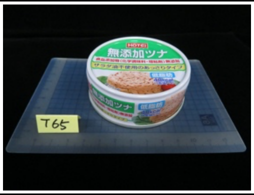			
B1B	B1C	B1D	B2E
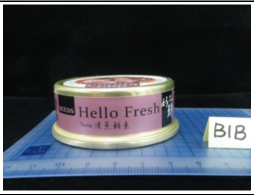	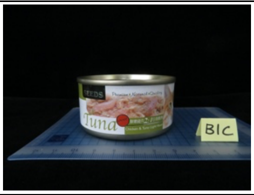	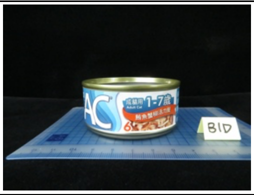	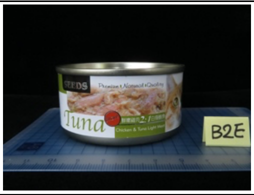
B2F	B2G	B3A	B3B
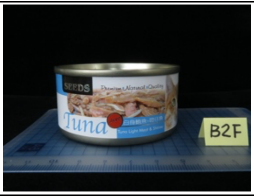	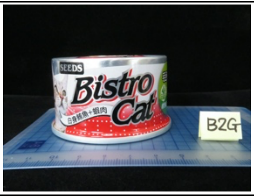	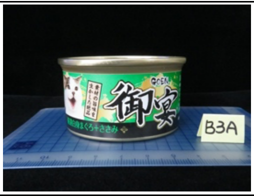	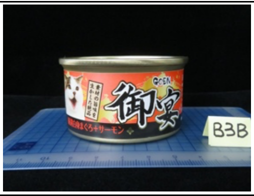
B3F	B3H	C1A	C1B
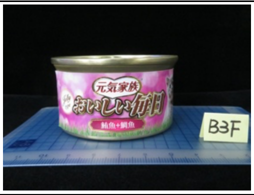	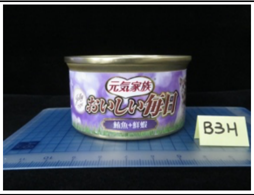	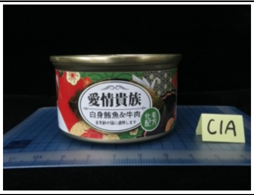	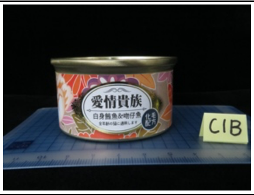
C2B	C2C	C4A	C5A
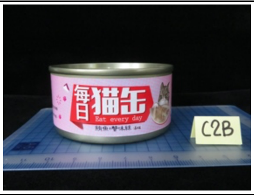	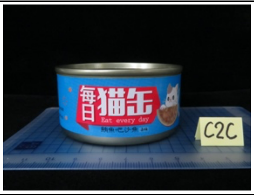	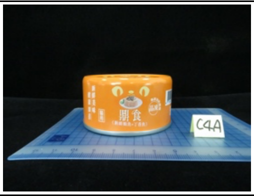	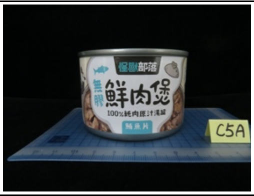
C4C	D1B	D1C	D2B

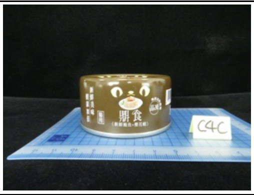	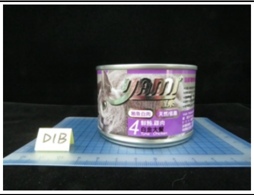	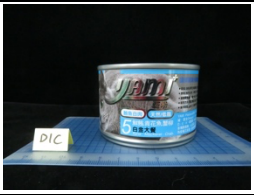	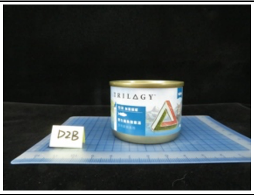
D2C	D2G	E1E	E2C
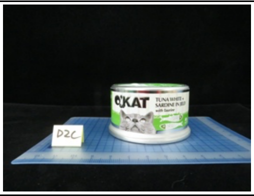	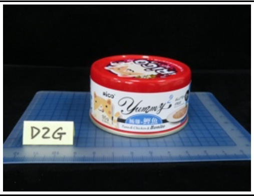	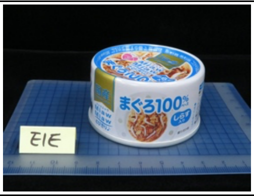	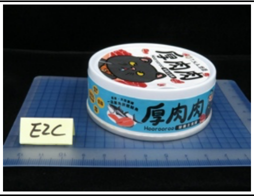
E2D			
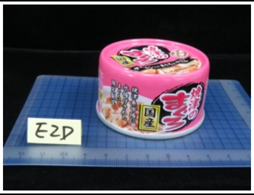			

Supplementary S2

Hap_A
ATGGAGCTTTAGACACCAAGGCATTTCATGTTAAACACCCCTGAATAAAGGACCAAACCAAAT
GAACCATGCCCCCATGTCTTTG

>Hap_B_bonito
ATGGAGCTTTAGACACTAAGGCATATCATGTTAAACACCCCTGAACAAAGGATTAAACCAAAT
GAATAATGCCCCCATGTCTTTG

>Hap_G_bonito
ATGGAGCTTTAGACACCAAGGCATATCATGTTAAACACCCCTAAACAAAGGGCTAAACCAAAT
GAATCATGCCCCCATGTCTTTG

>Hap_D_Thunnus
ATGGAGCTTTAGACACCAAGGCATATCATGTCAAACACCCCTAAACAAAGGACTAAACCAAAT
GAATCATACCCCCATGTCTTTG

>Hap_F_Thunnus
ATGGAGCTTTAGACACCAAGGCATATCATGTCAAACACCCCTAAACAAAGGACTAAACCAAAT
GAATCATGCCCCCATGTCTTTG

>Hap_H_Thunnus
ATGGAGCTTTAGACACCAAGGCATATCATGTTAAACACCCCTAAACAAAGGACTAAACCAAAT
GAATCATGCCCCCATGTCTTTG
>Hap_C_skipjack
ATGGAGCTTTAGACACCAAGGCATATCATGTTAAACACCCCTAAATAAAGGACCAAACCAAAT
GAATTATGCCCCCATGTCTTTG
>Hap_E_skipjack1
ATGGAGCTTTAGACACCAAGGCATATCATGTTAAACACCCCTAAATAAAGGACCAAACCAAAT
GAACTATGCCCCCATGTCTTTG

Supplementary S3
#Mega
!Title Tuna CR NJtree;

#FJ936139_Allothunnus_fallai
CTACATATATATTAACCAT-ATATATATAGTATTCAAGGTAC-ATTA-
ATGTATTATCAACATAACTAGTAATATACCATTCATATATCACCATTTACACTAAGGGTTACATAAA
GCTGTTTGACAAANTNCTCTC----GA-----------AT-GNNGAGCTA------
CGAGATTTAAGACCTAGCAC-
GAAACGNCCCTGGTCGAAGATATACCAAGCACTCAACACCCGATTAATCCTC-AG--AATCTTA
#D2G3
ATATCTATATATGGACCAT-ATACA-GTAATGTTCTAGG-AC-ATAC-
ATGTATTAAAACCATTACTAGTACTAAACCATTCATATATCAACA-
AACAATGAAGACTTACATAAACC---AT-ACAGATATATTCC----AGCATTCAAGTT-AA-
GTCAGGTAA-TTAAACGAGATTTAAGACCTACCAC-
AAACACTAAATCGTCTAAGCCATACCAAGTCTCC-TCATCC--CTGAAATCG-AGTAAATTTAA
#HQ853210_Thunnus_alalunga
ATAACTATATTTGAAACAT-ATATA-ATAATGCTTTAGG-AC-ATAT-
ATGTATTAAAACCATAACTAGTATTTAACCATTCATATGTCAACA-
TATCATGAAAACTTACATAAAGC---ATAACAATCATTCCCC----AACACTTTACTT-AT-
ATCACGTAA-TTAAACGAGATTTAAGACCTAACAC--
AAACCTAAATCGTCTAAGCCACACCAAGTCCCC-CCATCT--CTAAAATCT-GGTAAACTTAT
#HQ853211_Thunnus_alalunga
ATAACTATATTTGAAACAT-ATATA-ATAATGCTTTAGG-AC-ATAT-
ATGTATTAAAACCATAACTAGTATTTAACCATTCATATGTCAACA-
TATCATGAAAACTTACATAAAGC---ATAACAATCATTCCCC----AACACTTTACTT-AT-
ATCACGTAA-TTAAACGAGATTTAAGACCTAACAC--
AAACCTAAATCGTCTAAGCCACACCAAGTCCCC-CCATCT--CTAAAATCT-GGTAAACTTAT

#HQ853212_Thunnus_alalunga
ATAACTATATTTGAAACAT-ATATA-ATAATGCTTTAGG-AC-ATAT-
ATGTATTAAAACCATAACTAGTATTTAACCATTCATATGTCAACA-
TATCATGAAAACTTACATAAAGC---ATAACAATCATTCCCC----AACACTTTACTT-AT-
ATCACGTAA-TTAAACGAGATTTAAGACCTAACAC--
AAACCTAAATCGTCTAAGCCACACCAAGTCCCC-CCATCT--CTAAAATCT-GGTAAACTTAT
#AF390331_Thunnus_alalunga
ATAACTATATTTGAAACAT-ATATA-ATAATGCTTTAGG-AC-ATAT-
ATGTATTAAAACCATAACTAGTATTTAACCATTCATATGTCAACA-
TATCATGAAAACTTACATAAAGC---ATAACAATCATTCCTC----AACACTTTACTT-AT-
ATCACGTAA-TTAAACGAGATTTAAGACCTAACAC--
AAACCTAAATCGTCTAAGCCACACCAAGTCCCA-CCATCT--CTAAAATCT-GGTAAACTTAT
#AF390256_Thunnus_alalunga
ATAACTATATTTGAAACAT-ATATA-ATAATGCTTTAGG-AC-ATAT-
ATGTATTAAAACCATAACTAGTATTTAACCATTCATATGTCAACA-
TATCATGAAAACTTACATAAAAC---ATAACAACCATCTCCC----AACACTTTATTT-AT-
ATCACGTAA-TTAGACGAGATTTAAGACCTAACAT--
AAACCTAAATCGTCTAAGCCATACCAAGTCCCC-TCATCT--CTAAAATCT-AGTAAACTTAT
#AF390425_Thunnus_thynnus
ATAACTATATTTGAAACAT-ATATA-ATAATGCTTTAGG-AC-ATGT-
ATGTATTAAAACCATAACTAGTATTTAACCATTCATATGTCAACA-
TATCATGAAGACTTACATAAAAC---ATAGCAACCATCTCCC----AACACTTTATTA-AT-
ATCATGTAA-TTAGACGAGATTTAAGACCTAACAT--
AAACCTAAATCGTCTAAGCCATACCAAGTCCCC-TCATCT--CTAAAATCC-AGTAAACTTAT
#GQ438257_Thunnus_orientalis
ATAACTATATTTGAAACAT-AAATA-ATAATGCTTTAAG-AC-ATAT-
ATGTATTATAACCATAACTAGTACTTAACCATTCATATGTCAACA-
CACCATGAAGGCTTACATAAAGC---ATGACAATTATCTTTC----AACATCCTATTT-AT-
ATCACGTAA-TTAAACGAGATTTAAGATCTACCAT--
AAACCTAAATCGTCTAAGCCATACCAAGTCCCC-CCATCT--CTGAAATCT-GGTGAACTTAA
#GQ438256_Thunnus_orientalis
ATAACTATATTTGAAACAT-AAATA-ATAATGCTTTAAG-AC-ATAT-
ATGTATTATAACCATAACTAGTACTTAACCATTCATATGTCAACA-
TACCATGAAGACTTACATAAAGC---ATGATAATTATCTTTC----AACATCCTACCT-AA-
ATCACGTAA-TTAAACGAGATTTAAGACCTACCAT--
AAATCTAAATCGTCTAAGCCATACCAAGTCCCC-CCATCT--CTGAAATCT-CGTAAACTTAA
#AY650619_Thunnus_thynnus
ATAACTATATTTAAAACAT-AAATA-ATAATGCTTTGGG-AC-ATAT-

ATGTATTACAACCATAACTAGTACTTAACCACTCATATGTCAACA-
TATCATGAAGACTTACATAAAGC---ATGACAATTATCTTTC----AACATCCTACCT-AC-
ATCACGTAA-TTAAACGAGATTTAAGACCTACCAT--
AAACCTAAATCGTCTAAGCCATACCAAGTCCCC-TCATCT--CTGAAATCT-AGTAAACTTAA
#AB185022_Thunnus_orientalis
ATAACTATATTTGAAACAT-AAATA-ATAATGCTTTAAG-AC-ATAT-
ATGTATTATAACCATAACTAGTACTTAACCATTCATATGTCAACA-
TATCATGAAGACTTACATAAAGC---ATGACAATTATTCTTC----AACATCCTAGCT-AC-
ATCACGTAA-TTAAACGAGATTTAAGACCTACCAT--
AAACCTAAATCGTCTAAGCCATACCAAGTCCCC-TCATCT--CTGAAATCT-AGTAAACTTAA
#GU972555_Thunnus_orientalis
ATATTCATATATAAACCAT-ATATA-ATAATGTTTTAGG-AC-ATAT-
ATGTATTAAAACCATTACTAGTATTAAACCACTCATATGTCAATA-
AATAATGAAGATTTACATAAACC---AT-ACGAATAAGCCTC----AACATCCATCTG-AA-
CTCAGGCGA-TTAAACGAGATTTAAGACCTAACAT--
AAATCTAAATCGTCTAAGCCATACCAAGTCTCC-TCATCT--CTGACATCT-CGTAAACTTAA
#AY650414_Thunnus_thynnus
ATATTCATATATAGACCAT-ATATA-ATAATGTTTTAGG-AC-ATAC-
ATGTATTAAAACCATTACTAGTATTAAACCATTCATATGCCAACA-
AACAATGAAGATTTACATAAACC---AT-ACAAATAAATCTC----AACATTCATCTTGAA-
TTCAGGCGA-TTAAACGAGATTTAAGACCTAACAT--
AAATCTAAATCGTCTAAGCCATACCAAGTCCCC-TCATCT--CTGACATCT-CGTAAACTTAA
#GU972554_Thunnus_orientalis
ATATTCATATATGGACCAT-ATATA-ATAATGTTTTAGG-AC-ATAC-
ATGTATTAAAACCATTACTAGTATTAAACCATTCATATGTCAATA-
AATAATGAAGATTTACATAAACC---AT-ACAAATAAACCTC----AACATTCACCTTGTA-
TTCAGGCGA-TTAAACGAGATTTAAGACCTAACAT--
AAATCTAAATCGTCTAAGCCATACCAAGTCTCC-TCATCT--CTGACATCT-CGTAAACTTAA
#AY650412_Thunnus_thynnus
ATACTCATATATAGACCAT-ATATA-ATAATGTTTTAGG-AC-ATAT-
ATGTATTAAAACCATTACTAGTATTAAACCATTTATATGTCAATA-
AATAATGAAGATTTACATAAACC---AT-ACAAATAAACCTC----AACATTCATTTTGAA-
TTCAGGCGA-TTAAACGAGATTTAAGACCTAACAT--
AAATCTAAATCGTCTAAGCCATACCAAGTCTCC-TCATCT--CTGACATCT-CGTAAACTTAA
#AY650409_Thunnus_thynnus
ATATTCATATATAGACCAT-ATATA-ATAATGTTTTAGG-AC-ATAT-
ATGTATTAAAACCATTACTAGTATTAAACCATTCATATGTCAATA-
AATAATGAAGATTTACATAAACC---AT-ACAAATAAACCTC----AGCATTCGTCTTGAA-

TTCAGGCGA-TTAAACGAGATTTAAGACCTAACAT--
AAATCTAAATCGTCTAAGCCATACCAAGTCTCC-TCATCT--CTGACATCT-CGTAAACTTAA
#AY650410_Thunnus_thynnus
ATATTCATATATAGACCAT-ATATA-ATAATGTTTTAGG-AC-ATAT-
ATGTATTAAAACCATTACTAGTATTAAACCATTCATATGTCAATA-
AATAATGAAGATTTACATAAACC---AT-ACAAATAAACCTC----AACATTCATCTTGAA-
TTCAGGCGA-TTAAACGAGATTTAAGACCTAGCAT--
AAATCTAAATCGTCTAAGCCATACCAAGTCTCC-TCATCT--CTGACATCTACGTAAACTTAA
#HQ853219_Thunnus_obesus
ATACTTATATATTAACCAT-ATATA-ATAATGTTCTAGG-AC-ATAT-
ATGTATTAAAACCATTACTAGTATTAAACCATTCATATGTCAACA-
AACAATGAAGGTTTACATAAACC---AT-ACAGACATATCCC----AACATCCATACT-AA-
GTCAAGTAA-TTAAACGAAATTTAAGACCTACCAC-
AAACACTAAATCGTCTAAGCCAAACCAAGTCCCA-TCATCT--CTAAAATCG-AGTAAATTTAA
#HQ853217_Thunnus_obesus
ATACTTATATATTAACCAT-ATATA-ATAATGTTCTAGG-AC-ATAT-
ATGTATTAAAACCATTACTAGTATTAAACCATTCATATGTCAACA-
AACAATGAAGGTTTACATAAACC---AT-ACAGACATATCCC----AACATCCATACT-AA-
GTCAAGTAA-TTAAACGAAATTTAAGACCTACCAC-
AAACACTAAATCGTCTAAGCCAAACCAAGTCCCA-TCATCT--CTAAAATCG-AGTAAATTTAA
#DQ126429_Thunnus_obesus
ATACTTATATATTAACCAT-ATACA-ATAATGTTCTAGG-AC-ATAT-
ATGTATTAAAACCATTACTAGTATTAAACCATTCATATGTCAACA-
AACAATGAAGGTTTACATAAACC---AC-ACAGACATATCCC----AACATCCATACT-AA-
GTCAAGTAA-TTAAACGAAATTTAAGACCTACCAC-
AAACATTAAATCGTCTAAGCCAAACCAAGTCCCA-TCATCT--CTAAAATCG-AGTAAATTTAA
#DQ126364_Thunnus_obesus
ATACTTATATATTAACCAT-ATACA-ATAATGTTCTAGG-AC-ATAT-
ATGTATTAAAACCATTACTAGTATTAAACCATTCATATGTCAACA-
AACAATGAAGGTTTACATAAACC---AT-ACAGACATATCCC----AACATCCATACT-AA-
GTCAAGTAA-TTAAACGAAATTTAAGACCTACCAC-
AAACATTAAATCGTCTAAGCCAAACCAAGTCCCA-TCATCT--CTAAAATCG-AGTAAATTTAA
#DQ126626_Thunnus_obesus
ATACTTATATATTAACCAT-ATACA-ATAATGTTCTAGG-AC-ATAT-
ATGTATTAAAACCATTACTAGTATTAAACCATTCATATGTCAACA-
AACAATGAAGGTTTACATAAACC---AT-ACAGACATATCCC----AACATCCATACT-AA-
GTCAAGTAA-TTAAACGAAATTTAAGACCTACCAC-
AAACACTAAATCGTCTAAGCCAAACCAAGTCCCA-TCATCT--CTAAAATCG-AGTAAATTTAA

#HQ853218_Thunnus_obesus
ATACTTATATATTAACCAT-ATACA-ATAATGTTCTAGG-AC-ATAT-
ATGTATTAAAACCATTACTAGTATTAAACCATTCATATGTCAACA-
AACAATGAAGGTTTACATAAACC---AT-ACAGACATATCCC----AACATCCATACT-AA-
GTCAAGTAA-TTAAACGAAATTTAAGACCTACCAC-
AAACACTAAATCGTCTAAGCCAAACCAAGTCCCA-TCATCT--CTAAAATCG-AGTAAATTTAA
#HQ630707_Thunnus_maccoyii
ATATTTATATATCGACCAT-ACATA-ATAATGCTTTAGG-AC-ATAT-
ATGTATTAAAACCATTACTAGTATTAAACCATTCATATGTCAACA-
AACAACGAAGATTTACATAAACC---AT-ACAGATAAACTCC----AACATTCTCTTA-AA-
TTCAAGTAA-CTAAACGAGATTTAAGACCTAACAC--
AAATCTAAATCGTCTAAGCCATACCAAGTCTCC-TCATCC--CTGAGGTCT-GGTAAATTCAA
#HQ630711_Thunnus_tonggol
ATATCTATATATCGACCAT-ATATA-ATAATGCTTTAGG-AC-ATAT-
ATGTATTAAAACCATTACTAGTACTAAAACATTCATATGTCAACA-
AACAATGAAGACTTACATAAACC---AT-ACAGGTATATCTT----GACATTCAATCT-AA-
GTCAAGTAA-
TTAAACGAGATTTAAGACCTAGCATAAAGAACTAAATCGTCTAAGTTACACCAAGTATCC-
TCATCC--CTAAAATCA-AGTAAATTTAA
#T34
ATATCTATATATCGACCAT-ATATA-ATAATGCTTTAGG-AC-ATAT-
ATGTATTAAAACCATTACTAGTACTAAAACATTCATATGTCAACA-
AACAATGAAGACTTACATAAACC---AT-ACAGATATATCTT----GACATTCAATTT-AA-
GTCAAGTAA-
TTAGACGAGATTTAAGACCTAACATAAAAAACTAAATCGTCTAAGTTACACCAAGTATCC-
TCATCT--CTAAAATCA-AGTAAATTTAA
#HQ630706_Thunnus_atlanticus
ATATTCTTATATCGACCAT-ATATA-ATAATGCTTTAGG-AC-ATAT-
ATGTATTAAAACCATTACTAGTACTAAAGCATTCATATATCAACA-
AACCATGAAGATTTACATAAACC---AT-ACAAATATATCTT----AACATTCAACCT-AA-
GTCAAGTAA-TTAAACGAGATTTAAGACCTAACAT-
AAGAACTAAATCGTCTAAGTTATACCAAGTATCC-TCATTC--CTAAAATCA-AGCAAACTTAA
#T56
ATACTCATATATGGACCAT-ATATA-ATAATGCTTTAGG-AC-ATAC-
ATGTATTAAAACCATTACTAGTACTAAACCATTCATATATCAACA-
AATAATGAAGATTTACATAAACC---AT-ACAGGTATATCTT----AATATTCAATCT-AA-
GTCAAGTAA-TTAAACGAGATTTAAGACCTACCAT-
AACAACTAAATCGTCTAAGCCATACCAAGTATCC-CCATCC--CTAAAGTCA-GGTAAATTTAA

#T7_CR
ATACTCATATATCGACCAT-ATATA-ATAATGCTTTAGG-AC-ATAT-
ATGTATTAAAACCATTGCTAGTACTAAACCATTCATATGTCAACA-
AACAATGAAGATTTACATAAACC---AT-ACAGATATATCTT----AATATTCAATCT-AA-
GTCAAGTAA-TTAAACGAGATTTAAGACCTACCAT-
GACAACTAAATCGTCTAAGCCATACCAAGTATCC-CCATTC--CTAAAGTCA-AGTAAATTTAA
#DQ126345_Thunnus_albacares
ATACTCATATATCGACCAT-ATATA-ATAATGCTTTAGG-AC-ATAT-
ATGTATTAAAACCATTACTAGTACTAAACCATTCATATATCAACA-
AACAATGAAGATTTACATAAACC---AT-ACAGATATATCTT----AATATTCAATCT-AA-
GTCAAGTAA-TTAAACGAGATTTAAGACCTACCAT-
AATAACTAAATCGTCTAAGCCATACCAAGTACGC-TCATCC--CTAAAGTCA-GGTAAATTTAA
#DQ126342_Thunnus_albacares
ATACTCATATATCGACCAT-ATATA-ATAATGCTTTAGG-AC-ATAT-
ATGTATTAAAACCATTACTAGTACTAAACCATTCATATGTCAACA-
AACAATGAAGATTTACATAAACC---AT-ACAAATATATCTT----AATATTCAATCT-AA-
GTCAAGTAA-TTAGACGAGATTTAAGACCTACCAT-
AACAACTAAATCGTCTAAGCCATACCAAGTATCC-CCATCT--CTAAAGTCA-AGTAAATTTAA
#T1_CR
ATACTCATATATCGACCAT-ATATA-ATAATGCTTTAGG-AC-ATAT-
ATGTATTAAAACCATTACTAGTACTAAACCATTCATATGTCAACA-
AACAATGAAGATTTACATAAACC---AT-ACAGATATATCTT----AACATTCAATCT-AA-
GTCAAGTAA-TTAAACGAGATTTAAGACCTACCAT-
AACAACTAAATCGTCTAAGCCATACCAAGTATCC-TCATTC--CTAAAGTCA-AGCAAATTTAA
#HQ853213_Thunnus_albacares
ATACTCATATATCAACCAT-ATATA-ATAATGCTTTAGG-AC-ATAT-
ATGTATTAAAACCATTACTAGTACTAAACCATTCATATGTCAACA-
AACAATGAAGATTTACATAAACC---AT-ACAGATATATCTT----AATATTCAATCT-AA-
GTCAAGTAA-TTAAACGAGATTTAAGACCTACCAC-
AACAACTAAATCGTCTAAGCCATACCAAGTATCC-TCATTC--CTAAAGTCA-GGTAAATTTAA
#HQ853214_Thunnus_albacares
ATACTCATATATCAACCAT-ATATA-ATAATGCTTTAGG-AC-ATAT-
ATGTATTAAAACCATTACTAGTACTAAACCATTCATATGTCAACA-
AACAATGAAGATTTACATAAACC---AT-ACAGATATATCTT----AATATTCAATCT-AA-
GTCAAGTAA-TTAAACGAGATTTAAGACCTACCAC-
AACAACTAAATCGTCTAAGCCATACCAAGTATCC-TCATTC--CTAAAGTCA-GGTAAATTTAA
#HQ853216_Thunnus_albacares
ATACTCATATATCAACCAT-ATATA-ATAATGCTTTAGG-AC-ATAT-

ATGTATTAAAACCATTACTAGTACTAAACCATTCATATGTCAACA-
AACAATGAAGATTTACATAAACC---AT-ACAGATATATCTT----AACATTCAATCT-AA-
GTCAAGTAA-TTAAACGAGATTTAAGACCTACCAC-
AACAACTAAATCGTCTAAGCCATACCAAGTATCC-TCATTC--CTAAAGTCA-GGTAAATTTAA
#HQ853215_Thunnus_albacares
ATACTCATATATCAACCAT-ATATA-ATAATGCTTTAGG-AC-ATAT-
ATGTATTAAAACCATTACTAGTACTAAACCATTCATATGTCAACA-
AACAATGAAGATTTACATAAACC---AT-ACAGATATATCTT----AACATTCAATCT-AA-
GTCAAGTAA-TTAAACGAGATTTAAGACCTACCAC-
AACAACTAAATCGTCTAAGCCATACCAAGTATCC-TCATTC--CTAAAGTCA-GGTAAATTTAA
#HQ630697_Auxis_rochei
ATACATATATATTAAACAT-TTATA-ATAATGTTCCAGT-AC-ATTA-
ATGTACTATAACCATGTATAGTATTTAACCATTCAAGTTGTACTA-
GTTAACTAANGGTTACATAAACC---ATAACAA---TCTTTA----AATACAT-ATCT-AA-
TAAAGGGGA-CTAAGCGAGATTTAAGACCTACCACAAGAATCCAA--
CTACTAATATATACCAAGTACTCAACATCCCGCCGAACTCA----AAAGCTTA
#HQ630699_Auxis_thazard
ATACATATATATTAAACAT-ATATA-ATAGTGTTTTAGT-AC-ATTT-
ATGTATTATAACCATACATAGTATTAAACCATTCAGGTAATAATA-
ACTAACTAAGGGTTACATAAACC---ATATCAAGC---TTTA----AGTACATATACA-AT-
GATAGTAAG-ACTAACGAGATTTAAGACCGACCACAAAAATCCAA--
CTACTAATATATACCAAGTACCCACCATCTCGCCAAGATCA----AAAGCTTA
#KF058674_Auxis_thazard
ATACATATATATTAAACAT-ATATA-ATAATGTTTTAGT-AC-ATTT-
ATGTATTACAACCATACATAGTATTAAACCATTCAAGTAATAATA-
ATTAACTAAGGGTTACATAAACC---ATATCAAAC---TTTA----AGTACATATACA-AT-
GATAGTAAG-ACTAACGAGATTTAAGACCGACCACAAAAGTCCAA--
CTACTAATATATACCAAGTATCCACCATCTCGCCAAGATCA----AAAGCTTA
#KF058561_Auxis_thazard
ATACATATATATTAAACAT-ATATA-ATAATGTTTTAGT-AC-ATTT-
ATGTATTATAACCATACATAGTACTAAACCATTCAGGTAATAATA-
ATCAACTAAGGGTTACATAAACC---ATATCAAAC---TTTA----AGTACATATACA-AT-
GATAATAAG-ACTAACGAGATTTAAGACCGACCAC-AAAAGTCCAATTA-
CTAATATATACCAAGTACCCAGCATCTCGCCAAGATCA----AAAGCTTA
#KF058703_Auxis_thazard
ATACATATATATTAAACAT-ATATA-ATAATGTTTTAGT-AC-ATTC-
ATGTATTATAACCATATATAGTACTAAACCATTCAGGTAATGATA-
ATTAACTAAGGGTTACATAAACC---ATATCAAAC---TTTA----AGTACATATAC--AA-

TGATAGTAAGACTAACGAGATTTAAGACCGACCAC-AAAAGTCCAATTA-
CTAATATATACCAAGTACCCACCATCTCGCCAAGATCA----AAAGCTTA
#HQ630700_Euthynnus_affinis
ATACATATATATTAACCATAACATA-ATAATGCTCTAGG-ACAATAC-
ATGTATTATAACCATTTATAGTATTAAACCATTAATACAGCACCATATAACCTAAGGGTTACATAA
ACC---AT-ATAAAGAA---------
GACATACAATAGAAAGGTTTGATAGACTAGGCGAGATTTAAGACCGACCACGAAACGTCCAA
ACTCCTAAGATATACCAAGTATTCAACATCTCATAACACTCA----AAAGCTTA
#AY899503_Katsuwonus_pelamis
ATACATATATATTAACCAT-ATACA-ATAATGTTGAAGG-AC-
ATATAATGTATTAAAACCATTAATAGTACTAAACCATTAATACAGCACCATAATAACTAAGGGTTA
CATAAACC---AT-GCTATTGTTAGTCGGACAATACTTAATAT---------
GGAGGCCAGGCGAGATTTAAGACCTACCAC-
GAAAACTCACACTCCTAATATATACCAAGTACTCAACATCT--CGTCAGTCTTAACAAGCTTAA
#AY899420_Katsuwonus_pelamis_isolate
ATACATATATATTAACCAT-ATACA-ATAATGTTGAAGG-AC-
ATATAATGTATTAAAACCATTGATAGTACTAAACCATTAATATAACACCATAATAACTAAGGGTTA
CATAAACC---AT-ATCATTACCAATC---GGACATACG--TTAAT-ATAAAGA---
CTGGACGAGATTTAAGACCTACCAC-
AAGAACTCACACGCCTAATATATACCAAGCACTCAACATCT--CGTCAACTT-AGACAAGCTCA
#AY899421_Katsuwonus_pelamis
ATACATATATATTAACCAT-ATATA-ATAATGTTGAAGG-AC-
ATATAATGTATTAAAACCATTAATAGTACTAAACCATTAACATAGCACCATATTAATTAAGGGTTA
CATAAACC---AT-ACAACTGCTAATCGGACAATACTTAATAT-AT-G------GA-
CTAGACGAGATTTAAGACCTACCAC-
AAGAACTGACACGCCTAATATATACCAAGTATCCAACATCT--CATCAACTTAGACAAGCTTAA
#AY899457_Katsuwonus_pelamis
ATACATATATATTAACCAT-ATATA-ATAATGTTGAAGG-AC-
ATACAATGTATTAAAACCATTAATAGCATTAAACCATTAACATAGCACCATATTAACTAAGGGTTA
CATAAACC---AT-ATGACTGCTAGTTGGACAATACTCAATAT-AA--------
AGGCCAGGCGAGATTTAAGACCTACCAC-
AAGAACTCACACGCCTAATATATACCAAGTATCCACCATCT--CGTCAACTTGAACAAGCTTAA
